# Agricultural input modifies trophic niche and basal energy source of a top predator across human-modified landscapes

**DOI:** 10.3389/fevo.2023.1053535

**Published:** 2023-06-01

**Authors:** André C. Pereira, Christy J. Mancuso, Seth D. Newsome, Gabriela B. Nardoto, Guarino R. Colli

**Affiliations:** 1 Departamento de Zoología, Instituto de Ciências Biológicas, Universidade de Brasília, Brasília, Brazil; 2 Departamento de Ecología, Instituto de Ciências Biológicas, Universidade de Brasília, Brasília, Brazil; 3 Department of Biology, University of New Mexico, Albuquerque, NM, United States

**Keywords:** Araguaia floodplain, compound-specific stable isotope analysis, crocodilian, essential amino acids, hierarchical Bayesian modeling, pasture, spatial analysis

## Abstract

Land-use conversion and resulting habitat fragmentation can affect the source(s) of primary productivity that fuels food webs and alter their structure in ways that leads to biodiversity loss. We investigated the effects of landscape modification on food webs in the Araguaia River floodplain in central Brazil using the top predator, and indicator species *Caiman crocodilus* (Crocodilia, Alligatoridae). We measured carbon ($\delta^{13}C$) and nitrogen ($\delta^{15}N$) isotope values of three tissues with different isotopic incorporation rates to evaluate spatial and temporal changes in caiman isotopic niche width with hierarchical Bayesian models that accounted for habitat use, intraspecific trait variation (sex and body size), and landscape attributes (composition and configuration). We also measured $\delta^{13}C$ values of essential amino acids to assess if different primary producers are fueling aquatic food webs in natural and anthropogenic areas. Spatial analysis showed that caiman in agricultural areas had larger isotopic niche widths, which likely reflects some use of terrestrial resources in environments dominated by C_4_ plants. Patterns in $\delta^{13}C$ values among essential amino acids were clearly different between natural and anthropogenic habitats. Overall, our findings suggest that caimans can persist in heterogeneous landscapes fueled by natural and agricultural energy sources of energy, which has implications for effectively managing such landscapes to maximize biodiversity.

## 1. Introduction

Agribusiness requires extensive areas to meet human global food demand, compromising natural biodiversity and ecosystem processes (Phalan et al., 2013; Laurance et al., 2014). Floodplains provide fertile soils and water for agricultural activities but suffer intense habitat degradation and loss that impact terrestrial and aquatic ecosystems (Allan, 2004; Best, 2018). These changes occur in wetland ecosystems characterized by high complexity, productivity, and functionality that support unique and rich biodiversity and provide ecosystem goods and services (Millennium Ecosystem Assessment, 2005; Maltby and Baker, 2009). The middle Araguaia River floodplain in central Brazil, a region rich in natural communities and ecosystem processes, faces rapid conversion of native vegetation and waterbody management (pumping irrigation and damming) to supply agricultural demand and development primarily for soybean and rice production and livestock (Hunke et al., 2014; Companhia Nacional de Abastecimento [CONAB], 2015; Oliveira et al., 2015; Garcia et al., 2017; Araújo et al., 2019). Efforts to adopt sustainable practices in land and water management play an essential role in the ecological integrity of floodplain ecosystems like the Araguaia River, contributing to the conservation of biodiversity and ecosystem processes and reducing the negative impacts of anthropogenic alterations (Laurance et al., 2014; Leal et al., 2020).

Biodiversity patterns and food web dynamics are affected by the complexities of landscape modification (in the dimensions of extension, composition, and configuration) owing to the loss and fragmentation of natural habitats and alteration in the matrix of unsuitable habitats (Fischer and Lindenmayer, 2007; Haddad et al., 2015; Liao et al., 2017b). Landscape attributes, such as habitat configuration, size, and quality are determinants of population distribution and dynamics, and variation in these factors can even cause the local extinction of populations (Fahrig, 2003; Ewers and Didham, 2006). Matrix quality and type also play pivotal roles in population dynamics through factors such as permeability, hostility, disturbance, and resource availability (Quesnelle et al., 2015). However, matrix quality varies spatially and temporally for biodiversity (Driscoll et al., 2013). Because matrix and habitat quality are perceived at the species level, species traits (e.g., dispersal ability, habitat specialization, trophic level, and feeding behavior) are critically important for their persistence in fragmented landscapes (Ewers and Didham, 2006). Landscape simplification acts as an ecological filter and drives biotic homogenization of biodiversity abundance and richness, where restriction to habitat and resource availability favors species with ecological plasticity, whereas sensitive and specialist species are eliminated (Newbold et al., 2015; Le Provost et al., 2020). Such changes severely affect ecological processes such as productivity, functioning, stability, resilience, and resistance (Scheffer et al., 2001; Duffy et al., 2007; Hooper et al., 2012).

Stable isotope analysis (SIA) of consumer tissues offers an integrated analytical assessment of biochemical cycles, food web dynamics, and trophic niche information for organisms (Newsome et al., 2007; Crawford et al., 2008). Carbon isotope ($\delta^{13}C$) analysis can trace the basal carbon source(s) that fuel food webs while nitrogen isotope ($\delta^{15}N$) analysis is typically used to estimate trophic position and food chain length owing to predictable isotopic enrichment with each trophic transfer (Ben-David and Flaherty, 2012). An organism’s isotopic niche—a bidimensional *δ*-space (Newsome et al., 2007)—can be used as a proxy for niche variation related to ecological traits (e.g., body size and sex) that lead to differences in resource exploitation, ecosystem or habitat use, and trophic position (Marques et al., 2013; Nifong et al., 2015). In addition, SIA can reveal ecological responses to anthropogenic disturbances. For example, agricultural matrices (pastures or croplands) alter the nutrient dynamics and proportions of natural C_3_ autochthonous production and C_4_ allochthonous subsidies in aquatic food webs (Martinelli et al., 2007; Carvalho et al., 2015; Bentivoglio et al., 2016; Parreira de Castro et al., 2016). SIA can help link landscape modification with alterations in trophic dynamics that result in trophic downgrading, niche collapse, low niche redundancy, high niche overlap, homogenization of energy flow, and niche shifts (Layman et al., 2007; Resasco et al., 2017; Magioli et al., 2019; Price et al., 2019; Burdon et al., 2020).

Furthermore, spatial extension mediates the spatial heterogeneity of stable isotope ratios in ecosystems, including under small-scale and human influence (Zambrano et al., 2010; Doi et al., 2013; Merlo-Galeazzi and Zambrano, 2014) that can directly affect the isotopic niche (Ceia et al., 2014; Reddin et al., 2018). In general, landscape attributes are neglected in spatial food web models to elucidate the functioning mechanisms, especially in ongoing landscape modification worldwide (Pillai et al., 2011; Liao et al., 2017a). Ignoring spatial isotopic variability could lead to misinterpretation and potentially mask the impacts of anthropogenic disturbances. For example, SIA-based assessments of the impacts of anthropogenic disturbances in aquatic ecosystems often make inferences from categorical or disturbance gradient designs using dispersal-limited top predators, such as fishes (Carvalho et al., 2015; Price et al., 2019; Burdon et al., 2020). Such approaches are inappropriate for top predators in wetland ecosystems, which are often large-bodied and highly mobile species that utilize a generalist feeding behavior. For example, crocodilians explore all waterbody habitats and have population dynamics according to landscape attributes, exerting ecological influence on aquatic and adjacent terrestrial food webs (Somaweera et al., 2020). Thus, spatial analysis and landscape-level patterns (amount, composition, configuration of habitats, and matrix types) can integrate SIA data from top predators to provide critical information and context about spatial ecological processes in the floodplains (Wang et al., 2014; Riva and Nielsen, 2020).

Additionally, the limitations of studies using only bulk tissue SIA can make it challenging to identify the type(s) of primary production fueling aquatic or terrestrial food webs (Finlay and Kendall, 2008; Boecklen et al., 2011; Zaia Alves et al., 2017). The primary limitation is that algal-derived aquatic and plant-derived terrestrial primary producers often show overlap in their $\delta^{13}C$ composition (Whiteman et al., 2019). An emerging alternative approach is measuring the $\delta^{13}C$ values of essential amino acids. Plants and algae have distinct patterns in essential amino acid $\delta^{13}C$ values ($\delta^{13}C$_EAA_) due to differences in the way(s) each producer type synthesizes these compounds (Larsen et al., 2009, 2013; Besser et al., 2022). Most eukaryotic consumers cannot synthesize essential amino acids *de novo* and must route these compounds directly from the protein in their food, leading to minimal isotope alteration of essential amino acids as they are passed up the food chain (McMahon et al., 2015; Whiteman et al., 2019; Manlick and Newsome, 2022). Applying this approach to top consumers can identify the basal source(s) of energy that fuels the food webs they rely on and examine potential human-induced shifts in energy sources (Thorp and Bowes, 2016; Bowes et al., 2019), especially in landscapes heavily influenced by agriculture.

Here, we combined landscape attributes, species intraspecific traits, and isotope-based estimates of the trophic ecology of *Caiman crocodilus* (spectacled caiman) to investigate the anthropogenic impacts of landscape modification on the food webs of the Araguaia floodplain using a spatially explicit approach. *Caiman crocodilus* (Crocodylia, Aligatoridae) is an indicator species and a model organism for detecting and monitoring environmental impacts in the Araguaia floodplain owing to its high detectability and seasonal and ontogenetic movements across a variety of terrestrial and aquatic habitats (Rosenblatt et al., 2013; Somaweera et al., 2020; Pereira and Colli, 2022). We assessed (1) the effects of intraspecific traits of sex, ontogeny, and habitat use based on the $\delta^{13}C$ and $\delta^{15}N$ isotopes values of *C. crocodilus* tissues; (2) the influence of landscape attributes (land-use composition and wetland configuration) on the isotopic niche of *C. crocodilus*; and (3) the influence of crop-derived (rice and soybean) energy on the food webs utilized by *C. crocodilus* in human-modified environments.

## 2. Materials and methods

### 2.1. Study area

**FIGURE 1:**
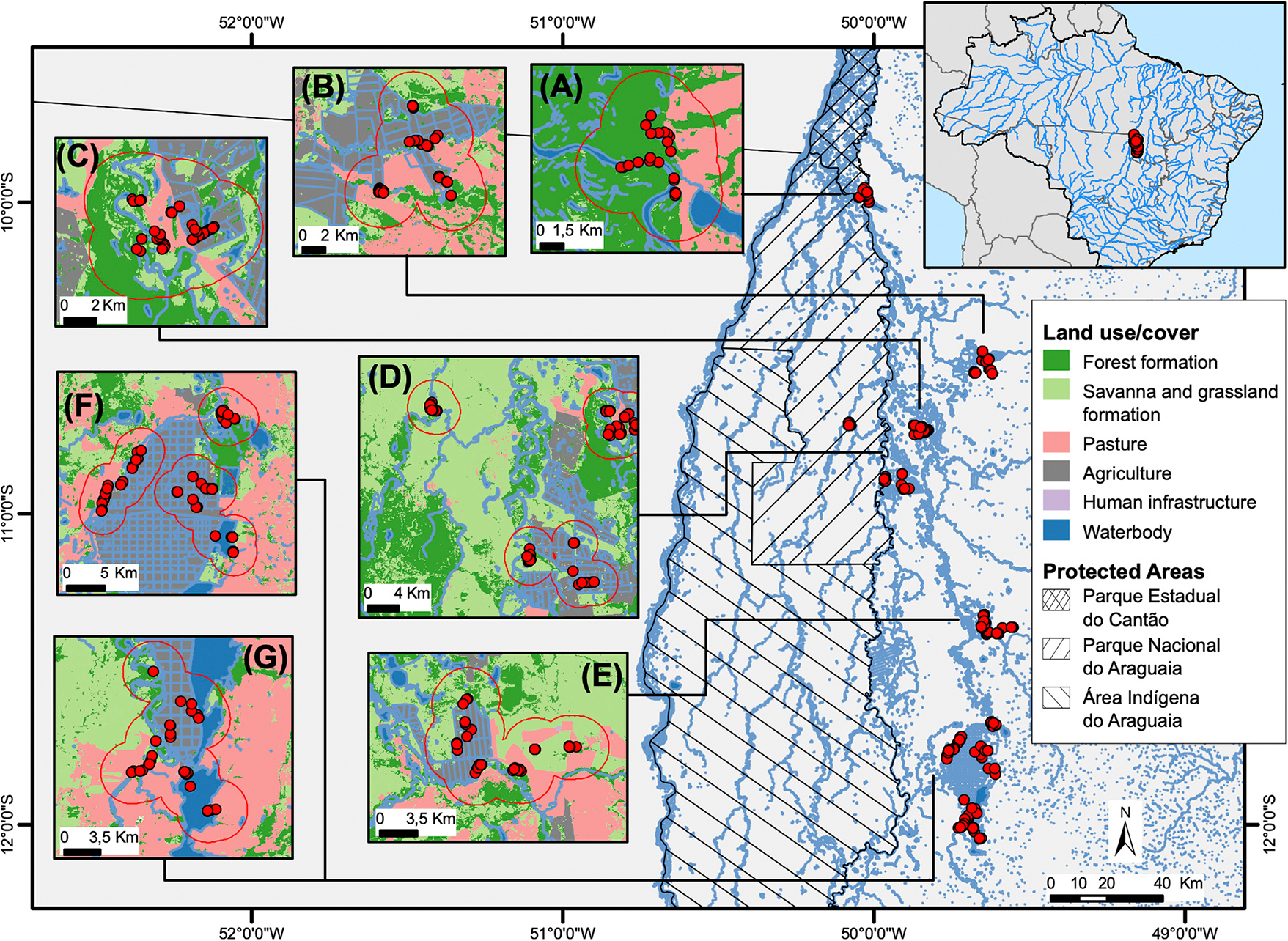
Location of seven sampling landscapes in the middle Araguaia River floodplain, Central Brazil: **(A)** Canguçu; **(B)** Cristalândia; **(C)** Lagoa; **(D)** Bananal; **(E)** Xavante; **(F)** Coopergran; **(G)** Cooperformoso. Inset boxes ordered from North to South. Land use classification, hydrograph, and protected areas in the region are denoted. Red points represent the position of captured caimans in each sampling site. Red lines indicate the maximum spatial region that include a 3 km buffer for landscape attributes estimates. The 3 km buffers were merged when they overlapped.

We conducted this study in the middle Araguaia River floodplain (Figure 1). The region is in a highly dynamic and complex Cerrado–Amazonia transitional zone in central Brazil (Marques E. Q. et al., 2020). The pronounced tropical wet-dry climate influences the flooding regime: the discharge increases from November to April (wet season) when the flood pulse can span approximately 88,000 km^2^ of surface area at maximum flood level and interconnects several waterbodies, and decreases during June and September (dry season), when waterbodies represent only 3.3% (2,930 km^2^) of the coverage area (Irion et al., 2016). The floodplain’s high spatial and temporal heterogeneity supports a rich and abundant biota, with many endemic and endangered species, sheltered in several protected areas and indigenous lands, including Bananal Island, RAMSAR site no. 624 (Ramsar Convention [RAMSAR], 2002). These protected areas are crucial in limiting the advance of fragmentation and land-use conversion (Carranza et al., 2014; Garcia et al., 2017).

However, this region is under sustained pressure from agricultural development funded by international and state programs because of the favorable topography and hydrology that has turned the floodplain into one of Brazil’s leading producers of irrigated rice (Fragoso et al., 2013; Companhia Nacional de Abastecimento [CONAB], 2015). The production is based on irrigated systems, where crops are cultivated according to the hydrological regime: rice in the wet season alternates with other crops (e.g., soybeans, beans, and watermelon) in the dry season (Oliveira et al., 2015). Similar to the entire Cerrado biome (Hunke et al., 2014; Dias et al., 2016), the Araguaia River Basin suffers sustained pressure from agricultural activities that have reduced native vegetation to less than 50% in the Upper Araguaia River (Ferreira et al., 2008; Coe et al., 2011) and changed the hydrogeomorphological dynamics owing to water damming, pumping, sedimentation, silting, erosion, and contamination (Latrubesse et al., 2009; Coe et al., 2011; Oliveira et al., 2015). A 26% reduction in native vegetation coverage was recorded between 1975 and 2013 in the middle Araguaia River floodplain, mainly driven by the expansion of pasturelands (Garcia et al., 2017). Currently, the land use pattern of the Cerrado is changing slowly from extensification to intensification of agricultural activities; however, pastureland coverage remains high (Dias et al., 2016).

### 2.2. Study design

We conducted fieldwork between July and September (dry season) in the years 2016 and 2018 in seven localities in five municipalities of Tocantins State (Figure 1), namely Bananal, Canguçu, Cristalândia, Cooperformoso, Coopergran, Xavante, and Lagoa (sampled in 2016). We sampled caimans in natural and anthropogenic habitats distributed across 32 sites under distinct land-use regimes, including inside and outside protected areas. Natural habitats include rivers and lakes derived from natural geomorphological processes in the Araguaia floodplain (Irion et al., 2016). Rivers comprise small and sizeable lotic water body tributaries of the Araguaia River, characterized by a sinuous water topology and natural riparian vegetation. Lakes include the small (0.5–5 ha) and large (>5 ha) lentic water bodies of diverse forms with riparian vegetation, but also large reservoirs created to supply water for agricultural activities. We defined anthropogenic habitats as waterbodies constructed for human activities within an agricultural matrix, such as artificial ponds (restricted to shallow waterbodies of <0.5 ha in area, created by soil excavation for livestock watering or fish farms and maintained by water complementation) and agricultural irrigation ditches—channelized drainages of permanent water flow inside agricultural fields—5–20 m wide and 0.5–2 m deep, with networks in a linear planform and right angles with vegetated or bare bank slopes (Davies et al., 2008; Herzon and Helenius, 2008; Biggs et al., 2016).

Migration and movement studies of South American crocodilians indicate a maximum movement distance of 20 km over 1–5 years (Gorzula, 1978; Ouboter and Nanhoe, 1988; Campos et al., 2006). Therefore, the sampling localities were at least 20 km apart, and sites within each locality were as far apart as possible. Therefore, we created circular buffer areas of 500 m, 1 km, and 3 km centered at each capture point to calculate the landscape metrics for the characterization, assessment, and measurement of human-modified landscapes. Based on home range studies, we considered buffer zones as a utilization area of caimans, which estimated ranges between 0.048 and 3.5 km^2^ (Ouboter and Nanhoe, 1988; Campos et al., 2006; Caut et al., 2019; Marques T. S. et al., 2020).

We assessed human land-use composition and wetland configuration through landscape metrics based on circular buffers of 500 m, 1 km, and 3 km centered at each animal in the site. First, we obtained land use/cover rasters from the MapBiomas Project (collection 4, 2016 and 2018).^1^ We grouped MapBiomas land-use classes into five categories: waterbody, forest, savanna (savanna, grassland, non-forest natural formation, and other non-forest natural formations), pasture (pasture and other non-vegetated areas), agriculture (annual and perennial crops), and urban (urban infrastructure). To improve water coverage assessment, we incorporated a hydrographic raster from a vector database acquired from Secretaria do Meio Ambiente e Recursos Hídricos of the State of Tocantins.^2^ Furthermore, we improved land use owing to differences between the supervised coverage in situ and the MapBiomas raster, reclassifying and redefining the topology guided by Landsat 8 satellite images for the same sampling period in 2016 and 2018 obtained from the Instituto Nacional de Pesquisas Espaciais–INPE (Brazilian Space Agency)^3^ using QGIS, version 3.12 (QGIS Development Team, 2020).

Second, we calculated the landscape metrics at the class and landscape levels for each buffer using the R package LANDSCAPEMETRICS (Hesselbarth et al., 2019). The landscape division index (LDI) was selected at the landscape level. At the class level, the metrics included only the proportion of classes (PCLASS) for all categories to describe the landscape composition. In contrast, the metrics at the patch level were restricted to water coverage to estimate wetland configuration: mean Euclidean nearest-neighbor distance (ENN), largest patch index (LPI), patch cohesion index (COHESION), and mean patch area (MPA). Such landscape metrics reflect the aspects of proportion, isolation, patch dominance, aggregation, physical connectivity, and landscape fragmentation (McGarigal and Marks, 1995; Jaeger, 2000).

Third, we minimized multicollinearity among landscape metrics using the variance inflation factor with a maximum of 4 in the R package USDM (Naimi et al., 2014), resulting in the retention of 14 metrics with a maximum correlation of r = 0.67 among them (Supplementary Table 1). We then calculated the mean values of landscape metrics at each site, applied a log (x + 1) transformation, and standardized the values around the mean with one standard deviation for posterior analyses.

### 2.3. Caiman sampling

We sampled each locality only once, staying between 4 and 7 days to perform captures, where we visited the sampled sites up to four times. We captured 275 caimans with sampling effort per site ranging from 6 to 14 animals and a sex ratio (male:female) of 2:1 (Supplementary Table 2). We captured caimans through nocturnal spotlight surveys with locking cable snares or by hand after locating the animals by eye reflection, with subsequent physical restraint of mouth and limbs with ropes and adhesive tape (Fitzgerald, 2012; Brien and Manolis, 2016). We recorded the snout-vent length (SVL; with a 0.1 cm precision tape), body mass (with 5, 10, or 50 kg spring scales, Scale Macro Line, Pesola Präzisionswaagen AG^®^, Schindellegi, Switzerland), and sex, determined by cloacal examination and palpation of the penis (Reed and Tucker, 2012). In addition, we individually marked *C. crocodilus* by notching tail scutes as a standardized numerical code and released them at the exact capture location after handling (Plummer and Ferner, 2012). We conducted this study under permits SISBIO #13324–6 and #57940–3 (issued by Instituto Chico Mendes de Conservação da Biodiversidade), FUNAI #08620.005147/2018–38 (Fundação Nacional do Índio), and CEUA-UnB #94/2017 (Comissão de Ética no Uso de Animais da Universidade de Brasília).

### 2.4. Bulk tissue $\delta^{13}C$ and $\delta^{15}N$ analysis

We collected claw (~5 mm fragments), tail muscle (1 g), and whole blood (~3 mL) samples from the captured animals for SIA (Beaupre et al., 2004; Fleming and Fontenot, 2015). Blood samples were obtained from the dorsal cervical sinus with a blood collection kit using 21G × 1” needles (25 × 8 mm) coupled to a 4 mL BD Vacutainer^®^ with lithium heparin anticoagulant, which showed no significant isotopic effect on plasma and red blood cell samples within 3 h (Kim and Koch, 2012). Blood samples were then centrifuged at 1,370 g for 60 s (OMEGA, Laborline^®^, São Paulo, Brazil) to separate and collect the plasma samples. In the field, we stored claw samples in plastic, while other tissue samples were stored at −80°C in a cryogenic liquid nitrogen container until preparation in the laboratory.

In the laboratory, claw and muscle samples were cleaned, and lipids were extracted with a 2:1 ratio of chloroform:methanol solvent solution for three washes for 2 h each (Post et al., 2007). Samples were then dried at 50°C and ground into a homogenous powder. Plasma tissue samples were freeze-dried for 24 h (Mod. E-C MicroModulyo, E-C Apparatus^®^) and stored at 20°C. Additionally, we collected and prepared crop samples with seeds from the Coopergran locality (rice: *n* = 10; soybean: *n* = 10). The samples were dried in an oven (60°C for 48 h) and ground into a homogenous powder. Finally, we weighed approximately 1–2 mg of each caiman sample and 2.0–2.5 mg of each crop sample and placed them into a 3 × 5 mm tin capsules for $\delta^{13}C$ and $\delta^{15}N$ analysis.

Carbon ($\delta^{13}C$) and nitrogen ($\delta^{15}N$) isotope values were determined by combustion using a Carlo Erba, CHN-1100 elemental analyzer coupled with a Thermo Finnigan Delta Plus isotope ratio mass spectrometer at the Laboratory of Isotope Ecology of the “Centro de Energia Nuclear na Agricultura” (CENA/Universidade de São Paulo), Piracicaba, São Paulo, Brazil. Based on the internationally recognized standard, the results were expressed in delta notation (δ) in parts per thousand $\left({\%}_{0}\right)$. The following equation was used:

$$\delta^{13}C\;\mathrm{or}\;\delta^{15}N=\left[\frac{R_{\mathrm{sample}}-R_{\mathrm{standard}}}{R_{\mathrm{standard}}}\right]\times 1000$$

where $R_{\text{sample}}$ and $R_{\text{standard}}$ represent the heavy and light isotope molar ratios (C13/C12 or N15/N14) of the sample and standard, respectively. The internationally accepted standards for $\delta^{13}C$ and $\delta^{15}N$ analysis is Vienna Pee Dee Belemnite (Vienna PDB; C13/C12 ratio = 0.01118) and atmospheric nitrogen (N15/N14 ratio = 0.0036765), respectively. Internal reference materials (USGS-42 and sugarcane leaves) were routinely interspersed with unknown samples. The mean within-run analytical precision for the internal reference materials was 0.2h for both $\delta^{13}C$ and δN15.

We also measured each sample’s weight percent carbon:nitrogen concentrations (C:N). Most samples had mean (±SD) C:N values within acceptable limits in plasma (3.3 ± 0.2), muscle (3.3 ± 1.9), and claw (2.9 ± 0.1) (Post et al., 2007), indicating little presence of lipids. However, 17 muscle samples had C:N values >4.0, suggesting a high lipid content (Post et al., 2007; Logan et al., 2008). To address this problem, we imputed the $\delta^{13}C$ values of these samples using the procedures described below in the Data Analysis subsection (Penone et al., 2014). We did not consider using lipid correction equations because such equations are species- and tissue-specific (Logan et al., 2008) and are not currently available for crocodilian tissues.

We evaluated resource use at multiple temporal and spatial scales using tissues with different isotopic incorporation rates that integrate diet over different periods (Crawford et al., 2008; Ben-David and Flaherty, 2012). Based on the tissue-specific isotopic incorporation rates available from a congener crocodilian species (*Caiman latirostris*), we assumed that plasma provides a relatively short timescale (~90 days), muscle reflects an intermediate timescale (130–190 days), and claws represent a relatively long timescale integrating >1 year of ecological information (Caut, 2013; Marques et al., 2014; Vander Zanden et al., 2015).

### 2.5. Essential amino acid (AA_ESS_) $\delta^{13}C$ analysis

We randomly selected muscle samples from 40 caimans for essential amino acid (AA_ESS_) $\delta^{13}C$ analysis. These samples were collected in both natural (lakes and river) and anthropogenic (ponds and ditches) habitats from four localities: 10 individuals from lakes (five from Canguçu and five from Bananal), 10 individuals from rivers (five from Canguçu and five from Bananal), 10 individuals from ponds (five from Coopergran and five from Cooperformoso), and 10 individuals from ditches (five from Coopergran and five from Cooperformoso). Descriptions of selected caiman populations are in Supplementary Table 3. Muscle samples were prepared for amino acid $\delta^{13}C$ analysis at the University of New Mexico Center for Stable Isotopes (Albuquerque, NM). A ~3–4 mg of lipid-extracted muscle sample was hydrolyzed to constituent amino acids in 1 ml of 6N HCl at 110°C for 20 h; tubes were flushed with N_2_ gas before sealing to prevent oxidation during hydrolysis. Amino acids were subsequently derivatized with 2-isopropanol and trifluoracetic acid (Silfer et al., 1991) and analyzed in duplicate to assess accuracy and precision. $\delta^{13}C$ measurements were made on a Thermo Scientific Delta Plus IRMS (Bremen, Germany) after samples were separated on a 60 m BPX5 column (SGE Analytical Science, Ringwood, Victoria, Australia) in a Thermo Scientific Trace 1310 gas chromatograph (GC, Bremen, Germany) and underwent combustion to CO_2_ in a ceramic reactor set at 1,000°C in a Thermo Scientific IsoLink II (Bremen, Germany).

For amino acid $\delta^{13}C$ measurements, we used a mixture of commercially available powdered amino acids (Sigma Aldrich, St. Louis, MO, USA) as a reference material derivatized and analyzed alongside each batch of unknown samples. All reference materials and unknown samples were processed and analyzed simultaneously with the same reagents and subject to the same protocols. $\delta^{13}C$ values for each underivatized amino acid were previously measured with a Costech 4,010 elemental analyzer coupled to a Thermo Scientific Delta V Plus IRMS (Breman, Germany). Like bulk tissue results, amino acid isotope data are reported using the standard *δ*-notation using the Vienna Pee Dee Belemnite (V-PDB) scale. We measured $\delta^{13}C$ values of six essential amino acids including threonine (Thr), valine (Val), leucine (Leu), isoleucine (Ile), phenylalanine (Phe), tyrosine (Tyr), and lysine (Lys). The average within-run standard deviation of $\delta^{13}C$ values of the in-house amino acid reference material ranged from 0.2h (isoleucine) to 0.6h (lysine); mean analytical precision across all six AA_ESS_ was and measurement procedures used for the AAh _ESS_ $\delta^{13}C$ analysis 0.4. We describe in Supplementary Appendix A the preparation (Whiteman et al., 2019).

### 2.6. Data analysis

We treated the missing values ($\delta^{13}C$ and $\delta^{15}N$ from eight individuals for muscle sample; *n* = 16) and $\delta^{13}C$ values for muscle samples with a C:N ratio > 4 (*n* = 17), representing 0.02% of all data (*n* = 1650), through imputation using the R package MISSFOREST (Stekhoven and Buühlmann, 2012). Imputation is a viable solution when missing data can introduce bias and lead to incorrect conclusions owing to the masking of biological patterns (Penone et al., 2014). MISSFOREST is a non-parametric method that relies on random forest algorithms to predict missing values (Stekhoven and Buühlmann, 2012). Performance is assessed using the normalized root mean squared error (NRMSE), where excellent performance leads to a value close to 0 (Stekhoven and Buühlmann, 2012). In our case, the NRMSE was 0.03%.

We estimated isotopic niche widths through the Bayesian standard ellipse area metric (SEA_B_; in h^2^) using the R package SIBER with its default settings (Jackson et al., 2011). SEA_B_ estimates were quantified at the site level for each tissue type. We also selected landscape metrics relevant to isotopic niche width using the R package BORUTA (Kursa and Rudnicki, 2010), a random forest-based selection method that identifies *all-relevant variables*. We used a *ntree* of 2,000, *maxRuns* of 2,000, and the default settings for the other parameters. We retained the landscape attributes with mean and normalized importance values above zero (*meanImp* and *normImp* > 0), obtained through the function *attStats* (Supplementary Tables 2, 4).

We implemented a hierarchical Bayesian approach to model the spatial variation in the (i) isotopic composition ($\delta^{13}C$ and $\delta^{15}N$) under the effects of intraspecific traits of sex, ontogeny, and habitat and (ii) the isotopic niche width of *C. crocodilus* under the effects of land-use composition and wetland configuration across landscapes in the Araguaia floodplain. Spatial hierarchical Bayesian models were structured through stochastic partial differential equations (SPDE) combined with the integrated nested Laplace approximation (INLA) algorithm using the R package R-INLA (Rue et al., 2009; Lindgren et al., 2011); thus, this approach accounted for the spatial dependency between sampling sites and the effects of selected predictors. We created models separately for each tissue, where the response variables were $\delta^{13}C$, $\delta^{15}N$, and isotopic niche width. The predictors were SVL, sex, habitat, and their interactions (for $\delta^{13}C$ and $\delta^{15}N$ models) or landscape attributes (for the isotopic niche width model). We applied backward stepwise procedures to INLA to obtain the best model using the *INLAstep* function in the R package INLAUTILS (Redding et al., 2017). We standardized the SVL around the mean with one standard deviation and applied an orthogonal contrast to categorical matrices using the *model.matrix* function.

For each model, we evaluated the performance of different mesh designs based on deviance information (DIC) and Watanabe-Akaike information (WAIC) criteria (Wang et al., 2018). We created five mesh designs using constrained refined Delaunay triangulation based on individual positions for the $\delta^{13}C$ and $\delta^{15}N$ models or sampling site locations for the isotopic niche width model by varying the sizes of triangles within and outside the sampled area (Supplementary Figure 1), attempting to minimize any boundary effects (Lindgren and Rue, 2015). Details about the models, representation of the spatial random fields, and descriptions of the posterior estimates of hyperparameters from the spatial hierarchical Bayesian approach are provided in Supplementary Appendix B.

We investigated differences in patterns of measured AA_ESS_ $\delta^{13}C$ values (or fingerprints) of caiman collected across habitats or localities. We performed linear discriminant analysis (LDA) using the R package MASS (Venables and Ripley, 2002) to discriminate between habitats or localities. We applied and examined the reclassification error rates using the leave-one-out cross-validation approach (Larsen et al., 2013). In LDA, we plotted the 95% confidence interval ellipses for each habitat or location, and the dataset was not standardized. All statistical tests were performed using R, version 3.6.1 (R Development Core Team, 2021).

## 3. Results

### 3.1. $\delta^{13}C$ and $\delta^{15}N$ models

The hierarchical Bayesian approach demonstrated that the mesh design performed differently in the $\delta^{13}C$ and $\delta^{15}N$ models as indicated by DIC and WAIC (Supplementary Tables 5, 6). The spatial structure of mesh 1 was the best for all tissues in $\delta^{13}C$ models, whereas mesh 1 (plasma and claw) and 5 (muscle) were the best for the $\delta^{15}N$ models. The isotopic models had similar random fields among tissues, with reduced spatial uncertainty in the regions of the sampled points (Supplementary Figures 2, 3).

**FIGURE 2:**
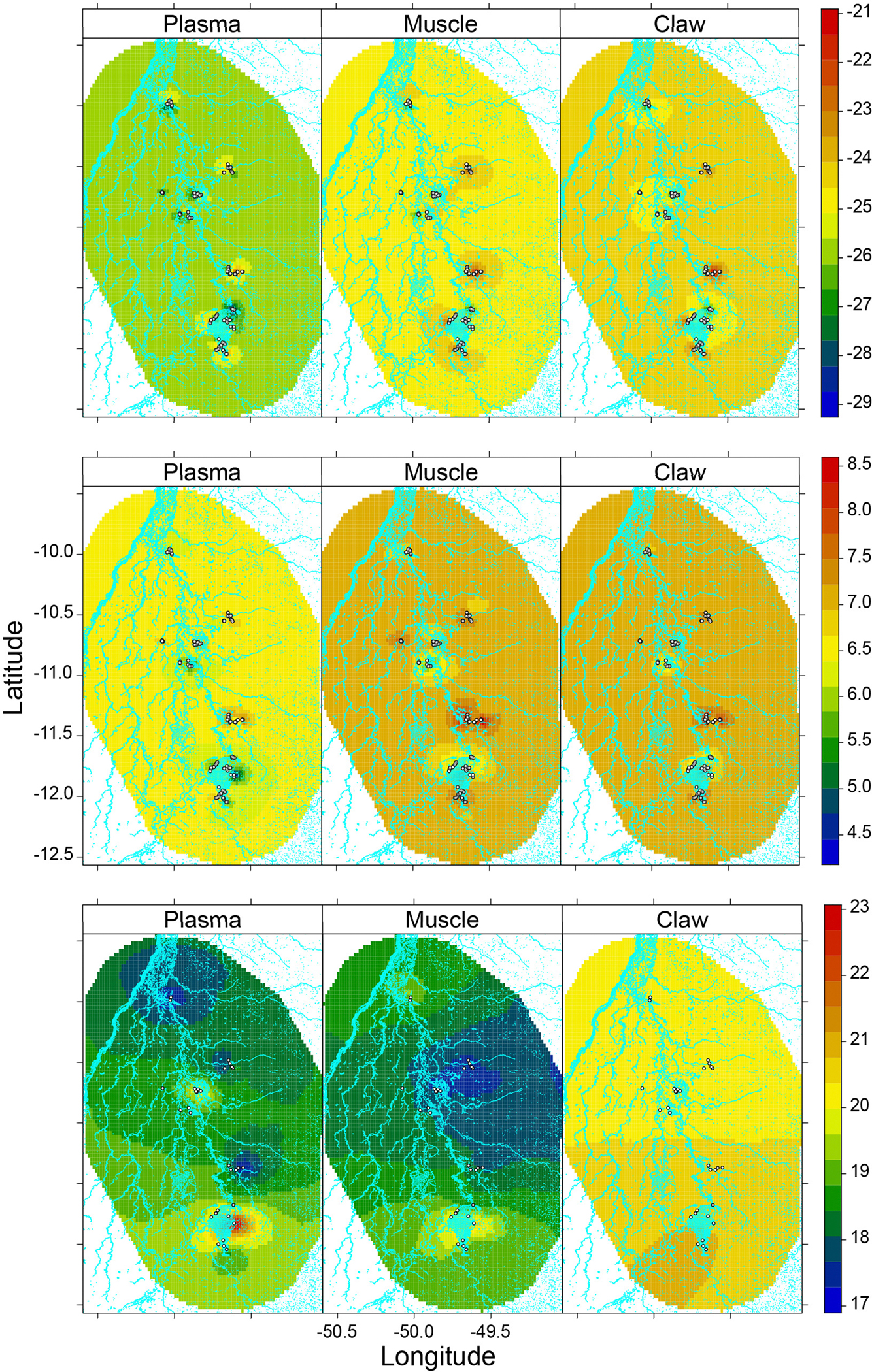
Predicted values from spatial hierarchical Bayesian best models for **(A)** $\delta^{13}C$, **(B)** $\delta^{15}N$, **(C)** isotopic niche width of *Caiman crocodilus* according to tissue across landscapes in the Araguaia floodplain. Hydrograph was depicted in light blue color in the frames and white points represent each sampled caiman **(A,B)** or sampling sites **(C)**. The colors indicate levels of mean $\delta^{13}C$ (%),$\delta^{15}N$ (%), isotopic niche width (%^2^) according to the associated legend. High values in bulk tissue $\delta^{13}C$ and $\delta^{15}N$ are related to anthropogenic habitats (e.g., irrigation systems) while surrounding natural habitats have lower values creating a spatial isotopic variability at landscape scales. Additionally, larger caiman isotopic niche widths were concentrated in the largest agricultural irrigation system and related to a greater proportion of pasture coverage. Some human-modified landscapes had similar niche widths as natural landscapes in the Araguaia floodplain, suggesting the same intensity of resource use in these populations across the landscape.

The spatial distribution of $\delta^{13}C$ and $\delta^{15}N$ values differed among tissues, i.e., the time window (Figures 2A, B). The predicted spatial distribution of $\delta^{13}C$ showed the lowest value in plasma and the highest in claw. In contrast, the predicted spatial distribution of $\delta^{15}N$ values showed little difference (~0.5h) among the tissues. The Xavante, Cooperformoso, and Coopergran localities had higher $\delta^{13}C$ and $\delta^{15}N$ values than other areas (Figures 2A, B and Supplementary Figures 4A, B).

In the $\delta^{13}C$ models, only habitat affected $\delta^{13}C$ in the plasma model (short timescale), with ponds having higher values than other habitats (Table 1). Models for muscle (intermediate timescale) and claw (long timescale) showed similar results, with significant effects of habitat, SVL, and habitat: sex: SVL interaction; muscle and claw collected in ponds had higher $\delta^{13}C$ values while ditches had lower $\delta^{13}C$ values in comparison to other habitats. SVL negatively affected muscle and claw $\delta^{13}C$ values, indicating that larger males positively influence the SVL–$\delta^{13}C$ relationship.

In the $\delta^{15}N$ models, only habitat significantly affected plasma $\delta^{15}N$ values, with ponds having lower values than other habitats (Table 2). Muscle $\delta^{15}N$ differed among habitats, with lakes having lower values than other habitats. Furthermore, habitat and sex influenced the SVL–$\delta^{15}N$ relationship, in which males in the ditch showed that $\delta^{15}N$ values decreased with SVL while in the pond, males showed that $\delta^{15}N$ values increased with SVL. Finally, claw $\delta^{15}N$ was affected by the SVL–$\delta^{15}N$ relationship owing to the habitat effect of the ditch, showing a negative trend. The same SVL–$\delta^{15}N$ relationship in the ditch differed according to sex, with males having a negative effect.

### 3.2. Effect of land use composition and wetland configuration on caiman isotopic niche width

We found similar DIC and WAIC values among mesh designs within each tissue model, suggesting that the structures had similar spatial dependencies in the hierarchical Bayesian approach for isotopic niche width (Supplementary Table 7). However, we selected the mesh structure with the lowest DIC and WAIC values: mesh 2 for plasma, mesh 5 for muscle, and mesh 1 for the claw. The spatial random fields of caiman isotopic niche widths varied according to tissue type (Supplementary Figures 2C, 3C). Plasma and claw random fields had low spatial dependence and reduced uncertainty across the Araguaia floodplain, whereas the muscle random field had high dependence and uncertainty, especially in the north and south of the study area.

The Boruta and INLA stepwise selection procedures retained only the proportion of pasture coverage and the fragmentation index for models of isotopic niche width (Table 3). The proportion of pasture coverage in the 500 m buffer affected the caiman isotopic niche width, with a positive effect in the plasma. The remaining predictors in their respective tissue models did not affect the caiman isotopic niche widths. The predicted isotopic niche width showed spatial variability across sites with a large range in plasma, intermediate range in muscle, and small range in claw (Figure 2C and Supplementary Figure 4C). Plasma isotopic niche widths were remarkably high in the Cooperformoso and Coopergran regions. For muscle, the central region of the study area had small isotopic niche widths, whereas the Cooperformoso and Coopergran regions maintained high values. Overall, the spatial distribution of isotopic niche width in the claws was higher than in other tissues, but it was distributed homogeneously across the Araguaia floodplain.

### 3.3. Essential amino acid (AA_ESS_) $\delta^{13}C$ analysis

**FIGURE 3:**
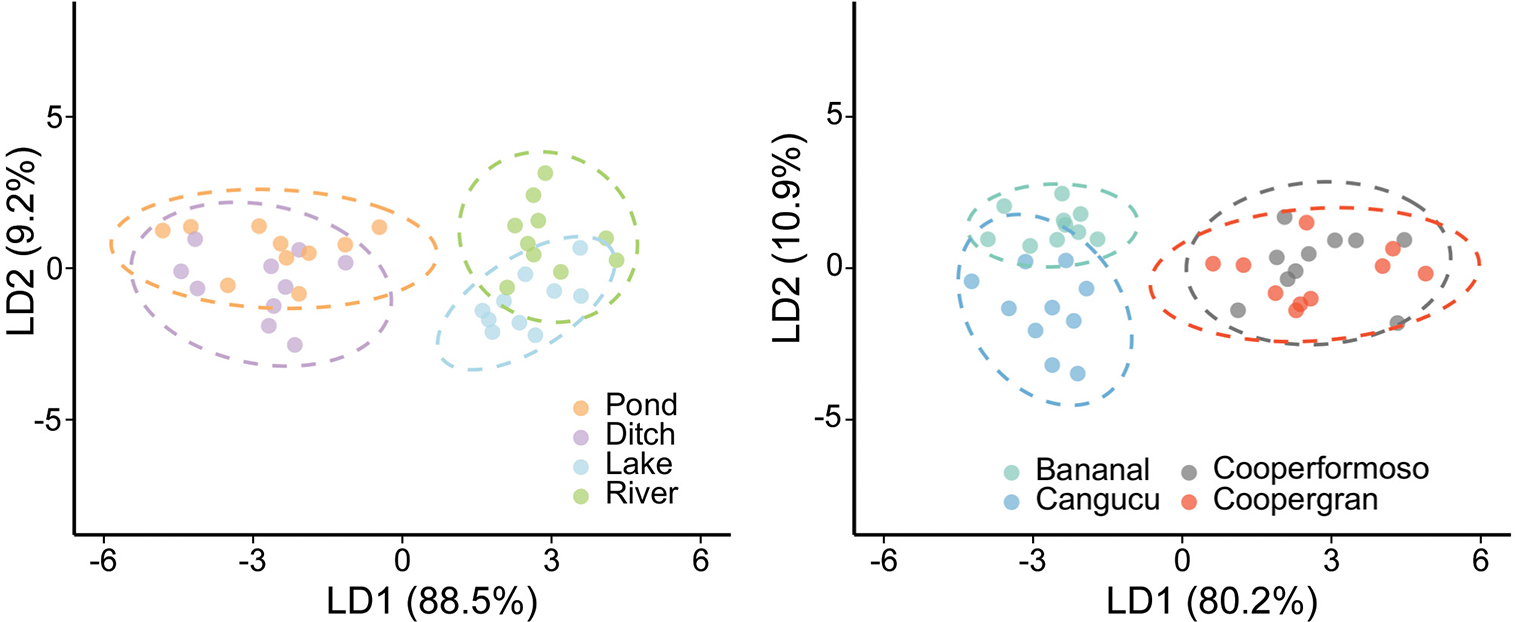
Multivariate discrimination based on AAESS $\delta^{13}C$ values of *Caiman crocodilus* according to habitat **(A)** and locality **(B)**. Ellipses indicate the 95% confidence interval region for classified groups of *C. crocodilus*. There is a distinction in basal carbon sources fueling food webs in natural versus anthropogenic habitats utilized by caiman, indicating an anthropogenic influence on energy and nutrient flow.

Linear discriminant analysis showed that essential amino acid (AA_ESS_) $\delta^{13}C$ patterns differed among habitats with an overall successful reclassification rate of 65%. Successful reclassification was 50% for ponds, 60% for ditches, 70% for lakes, and 80% for rivers (Supplementary Appendix C). The linear discriminant axes explained 88% (LD1) and 9% (LD2) of the overall variation among habitats, and the most informative coefficients were phenylalanine, leucine, and lysine $\delta^{13}C$ values. The LDA results showed a clear distinction between caiman sampled in natural (rivers and lakes) versus anthropogenic (ponds and ditches) habitats (Figure 3A).

Linear discriminant analysis also showed that AA_ESS_ $\delta^{13}C$ patterns differed among localities (Figure 3B), with an overall correct reclassification rate of 85%. Successful reclassification varied between localities: Canguçu (70%), Coopergran (80%), Cooperformoso (90%), and Bananal (100%) (Supplementary Appendix D). LD1 and LD2 explained 80 and 10% of the variation, respectively, and the most informative coefficients were for phenylalanine, leucine, and lysine $\delta^{13}C$ values.

## 4. Discussion

We showed that human-induced landscape modifications affect wetland food webs in the Araguaia floodplain. Analysis of spatial isotopic patterns showed that high values of $\delta^{13}C$ and $\delta^{15}N$ values, as well as large isotopic niche widths of *Caiman crocodilus* were associated with agricultural areas. Pasture coverage was the principal landscape feature that affected caiman niche width, with changes resulting from land-use conversion, habitat alteration, and fragmentation. Moreover, AA_ESS_ $\delta^{13}C$ analysis revealed that natural and anthropogenic habitats differed in basal carbon sources, indicating that crop-derived energy contributed to fuel caiman food webs in anthropogenic habitats.

Crocodilians are highly mobile top predators with opportunistic and generalist foraging strategies (Magnusson et al., 1987; Da Silveira and Magnusson, 1999; Somaweera et al., 2020). The diet varies with ontogeny, i.e., hatchlings feed primarily on aquatic and terrestrial invertebrates, whereas adults feed on vertebrates and fishes; flood pulse, with invertebrates predominating in the wet season and fishes in the dry season (Magnusson et al., 1987; Thorbjarnarson, 1993, 1997; Da Silveira and Magnusson, 1999); and sex, with mature females using different habitats and consumed resources during the nesting period for mature females (Barão-Nóbrega et al., 2016). Moreover, crocodilians can participate in aquatic and terrestrial food webs according to prey preferences and habitat use. Sympatric Amazonian crocodilians (*Paleosuchus trigonatus*, *P. palpebrosus*, *Melanosuchus niger*, and *Caiman crocodilus*) exhibit interspecific niche divergences based on the energy source, with more autochthonous sources in the floodplains over allochthonous inputs in the headwaters (Villamarín et al., 2017). We show that habitat, sex, and ontogeny regulate the strength and dynamics of their trophic interactions (Rosenblatt et al., 2013; Somaweera et al., 2020). Crocodilian studies show niche divergence through ontogenetic variation along stable isotopes or in the isotopic niches, which relates to reduced intraspecific competition (Radloff et al., 2012; Marques et al., 2013; Nifong et al., 2015; Caut et al., 2019). A previous study in the Araguaia region indicated that sex-related ontogenetic shifts drive isotopic niche partitioning in *Caiman crocodilus* that occupy similar habitats (Pereira et al., 2018). Such variations can be mediated by density-dependent mechanisms (such as social hierarchy and sexual dimorphism or nutritional and physiological requirements) to impose differences in the isotopic values, and niche segregation at the habitat and microhabitat level (Marques et al., 2013; Caut et al., 2019).

We observed marked landscape-scale spatial heterogeneity in $\delta^{13}C$ and $\delta^{15}N$ values of caimans from the Araguaia River floodplain. This variation is likely driven by spatial variation in the sources of primary production—aquatic versus terrestrial or natural versus agricultural across distinct habitats— and thus, to ecological processes and conditions across distinct habitats (Finlay and Kendall, 2008; Boecklen et al., 2011; Zaia Alves et al., 2017). Similar patterns in isotope variation across small spatial scales have been reported in artificial and natural freshwater ecosystems (Zambrano et al., 2010; Doi et al., 2013; Merlo-Galeazzi and Zambrano, 2014). Additionally, we found that caiman trophic niche width was influenced by pasture coverage in the Araguaia region, with high values clustered in the most extensive irrigation systems such as the Cooperformoso and Coopergran areas. The conversion of the floodplain to pasture changes the photosynthetic type of terrestrial production from C_3_ trees/shrubs to C_4_ grasses and alters soil hydro-physical properties that maximize the susceptibility of aquatic ecosystems to pasture inputs through erosion, sedimentation, and leaching processes, including a reduction in riparian vegetation (Latrubesse et al., 2009; Coe et al., 2011; Hunke et al., 2014). While (C_4_) grass fragments can enter the aquatic ecosystem and increase the $\delta^{13}C$ composition of dissolved inorganic carbon and particulate organic matter at the base of aquatic food webs (Martinelli et al., 2007), such resources are not easily assimilated by aquatic consumers, who instead favor higher quality autochthonous (algae) or allochthonous (C_3_ terrestrial) resources (Wantzen et al., 2010; Thorp and Bowes, 2016). C_4_-derived carbon from pasture or savanna can be introduced into caiman tissues by consuming insectivorous-omnivorous fishes or terrestrial invertebrate or vertebrate grazer prey (Wantzen et al., 2010). Our results show that caimans that use artificial ponds are highly susceptible to that allochthonous subsidy (Jardine et al., 2017).

Basal autochthonous (particulate organic matter and algae) and allochthonous (C_3_ and C_4_ plants) resources that fuel aquatic food webs adjacent to pasturelands can have highly variable $\delta^{13}C$ and $\delta^{15}N$ values, which can be identified and monitored via analysis of consumer tissues (García et al., 2017). Thus, variation in the proportion of pasture coverage can drive considerable alterations in basal resources, feeding behaviors, and isotopic niche sizes of consumers. Large-scale conversion of wetlands to pasture can be an irreversible change (in the sense of intangible recovery of the previous state), disrupting ecological processes that define food web structure and function (Fischer and Lindenmayer, 2007; Tscharntke et al., 2012; Haddad et al., 2015). The relevant association of the fragmentation index with the caiman isotopic niche suggests a chronic effect of landscape modification and habitat disturbance on terrestrial and aquatic food webs through land-use conversion, expanding an agricultural matrix over natural vegetation. Overall, the Cerrado biome suffers from a historical and constant pressure of pasturelands and cropland expansion (Barretto et al., 2013; Dias et al., 2016), including in the Araguaia River Basin (Ferreira et al., 2008; Coe et al., 2011; Garcia et al., 2017). The favorable climate, topography, and soil physical properties in the Araguaia floodplain linked to government incentives through technological, mechanical, and financial support have converged this region into an agricultural frontier (Fragoso et al., 2013; Phalan et al., 2013; Araújo et al., 2019). Although future agribusiness expansion can be reduced by agriculture intensification and new protected areas (Barretto et al., 2013; Carranza et al., 2014; Dias et al., 2016; Garcia et al., 2017), areas of natural vegetation will still be fragmented and converted to pasturelands or croplands, with species in the Cerrado facing a considerable challenge to persist (Strassburg et al., 2017; Lemes et al., 2019).

**FIGURE 4:**
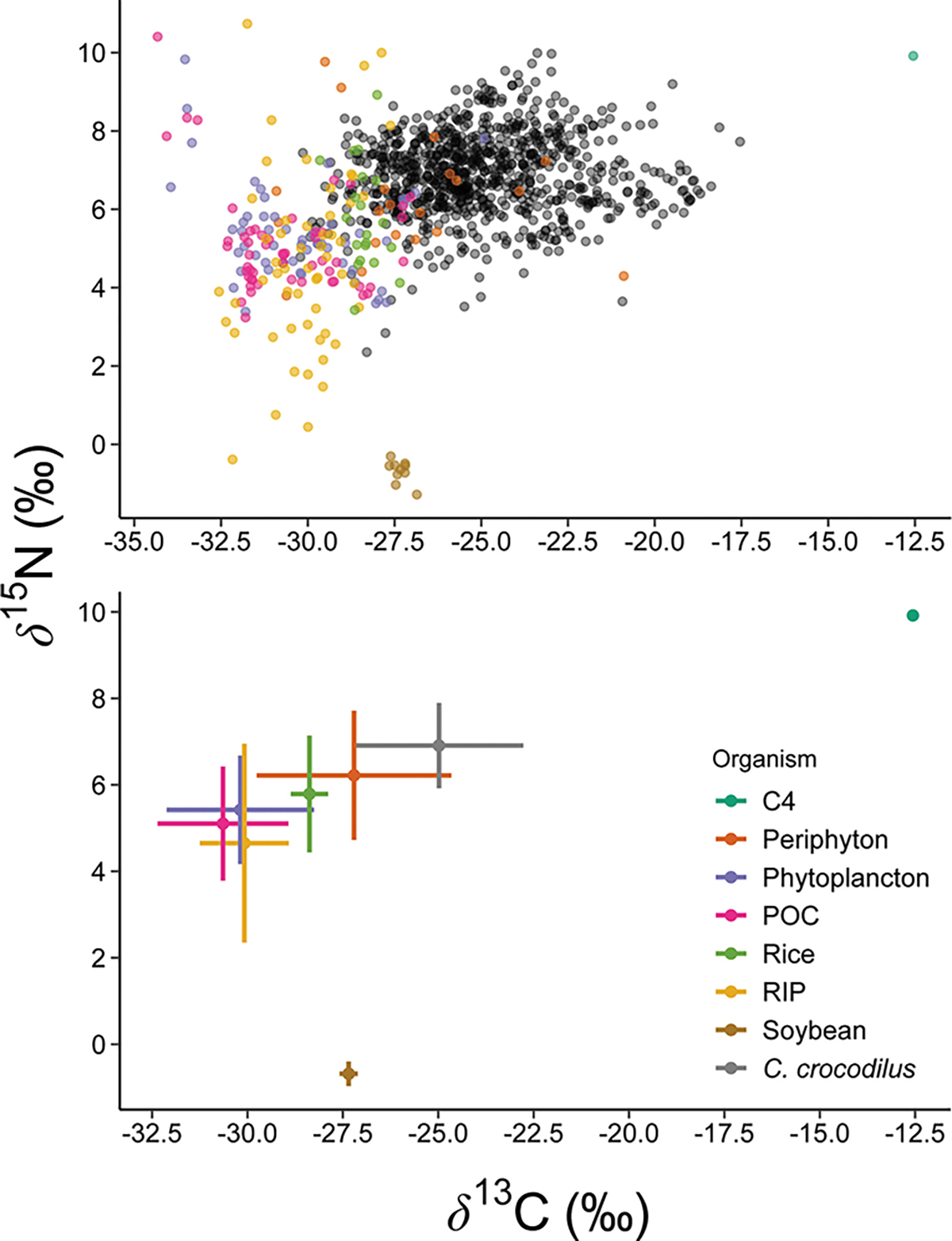
$\delta^{13}C$ and $\delta^{15}N$ values **(A)** and associated means ± SD **(B)** of basal sources in the Araguaia River Basin, crop samples (rice and soybean), and all caiman samples collected in our study. Remarkable isotopic variability in the bulk $\delta^{13}C$ and $\delta^{15}N$ occurs in basal sources, including with agricultural samples and caimans. Basal source isotopic data for primary producers is from Zaia Alves et al. (2017) and includes periphyton, phytoplankton, particulate organic carbon (POC), C_4_ terrestrial grass, and C3 riparian vegetation.

Bulk tissue stable isotope analysis shows high isotopic variability and overlap of $\delta^{13}C$ values between aquatic (algal) primary producers and the most common crops harvested in agricultural matrices (soybean and rice) in the Araguaia River floodplain, Figure 4 (Zaia Alves et al., 2017). However, essential amino acid $\delta^{13}C$ data identified a clear distinction in basal carbon sources fueling food webs in natural versus anthropogenic habitats utilized by caiman, indicating an anthropogenic influence on energy and nutrient flow. We hypothesize that the distinct AA_ESS_ $\delta^{13}C$ fingerprints between habitats or localities is driven by the incorporation of carbon from (C_3_) crops (soybeans and rice) which could have distinct AA_ESS_ $\delta^{13}C$ fingerprints in comparison to natural (C_3_) vegetation. We acknowledge that this hypothesis has not been rigorously tested. Alternatively, a recent study shows that C_3_ and C_4_ plants have distinct AA_ESS_ $\delta^{13}C$ fingerprints (Besser et al., 2022), so the patterns shown in Figure 3 could be primarily driven by greater incorporation of C_4_ resources caiman diets in anthropogenic habitats, ponds and ditches (Pereira et al., 2018; Pereira and Colli, 2022). Unfortunately, we cannot currently discriminate between these two explanations because AA_ESS_ $\delta^{13}C$ data are unavailable for local primary producers.

## 5. Conclusion

The spatially explicit Bayesian models approach employed here helps explore the relationship between landscape attributes and species responses that consider intraspecific variations and avoid dichotomic/categorical landscape evaluations (e.g., Resasco et al., 2017; Magioli et al., 2019) that do not reflect spatial variations and mechanisms that moderate the landscape use by organisms (Tscharntke et al., 2012; Wang et al., 2014; Riva and Nielsen, 2020). Landscape configuration drives food web structure and trophic interactions (Rooney et al., 2008; Pillai et al., 2011; Liao et al., 2017b). Realistic ecological responses to landscape alteration arise from considering species traits (e.g., trophic level, feeding behavior, body size, and dispersal ability) and species-oriented habitat perception (Ewers and Didham, 2006). These traits interact with landscape characteristics modeling species’ sensitivity and tolerance in the face of disturbance and determining the persistence in human-modified landscapes (Villard et al., 2014).

Our findings support evidence that a mixture of natural and anthropogenic (agricultural) energy can support top predators in highly modified landscapes in the Araguaia River floodplain. However, previous studies found that changes in trophic structure occur and energy channels can be lost in anthropic landscapes, making it unfeasible for a food web to support top predators in the long term (Layman et al., 2007; Liao et al., 2017b), triggering a trophic cascade with pronounced impacts on ecosystem resilience and resistance to disturbances (Scheffer et al., 2001; Duffy et al., 2007; Hooper et al., 2012). Our results show that the diversification of energy pathways (or channels) may stabilize the structure of food webs in some human modified environments. The fragmentation threshold for species extinction depends on community and landscape contexts (Villard et al., 2014; Liao et al., 2017a). Understanding the potential of anthropogenic landscapes to support biodiversity and ecological and conservation values relies on evaluating the key attributes of species, food webs, and ecosystem processes in the spatial context of landscape properties (Rooney et al., 2008). Our results emphasize that landscape modification can be reflected in the trophic niche of a semi-aquatic top predator, and provides new insights into how landscape fragmentation affects food web dynamics in a human-modified floodplain. These results enhance our understanding and contributing critical information to environmental policies, conservation planning, and land use management.

## Supplementary Material

Supplement

## Figures and Tables

**TABLE 1:** Posterior estimates (mean, SD, and 95% credibility interval) from spatial hierarchical Bayesian best models relating sex, snout-vent length (SVL), and habitat effects to δ^13^C values of *Caiman crocodilus* across landscapes in the Araguaia floodplain.

Tissue	Parameter	Mean	SD	Q_0.025_	Q_0.975_
Plasma	Intercept	−25.764	0.413	−26.609	−24.960
	Ditch	−0.174	0.336	−0.840	0.484
	Lake	−0.377	0.334	−1.039	0.274
	**Pond**	**1.200**	**0.361**	**0.498**	**1.915**
	Male	−0.103	0.115	−0.330	0.122
	SVL	−0.282	0.150	−0.576	0.012
	Ditch:Male	−0.027	0.169	−0.359	0.305
	Lake:Male	0.191	0.174	−0.150	0.532
	Ditch:SVL	−0.315	0.200	−0.708	0.078
	Male:SVL	−0.260	0.144	−0.544	0.023
	Ditch:Male:SVL	−0.216	0.200	−0.608	0.175
	Pond:Male:SVL	0.265	0.187	−0.102	0.632
Muscle	Intercept	−24.616	0.401	−25.446	−23.841
	**Ditch**	**−0.725**	**0.318**	**−1.354**	**−0.102**
	Lake	0.417	0.332	−0.241	1.066
	**Pond**	**0.851**	**0.341**	**0.187**	**1.530**
	**SVL**	**−0.451**	**0.149**	**−0.744**	**0.158**
	Ditch:Male	−0.170	0.185	−0.534	0.194
	Lake:Male	0.398	0.223	−0.040	0.835
	Pond:Male	−0.183	0.217	−0.609	0.243
	Ditch:SVL	−0.208	0.216	−0.632	0.216
	Lake:SVL	0.340	0.261	−0.174	0.853
	Male:SVL	−0.142	0.144	−0.425	0.142
	Ditch:Male:SVL	−0.381	0.213	−0.800	0.038
	**Lake:Male:SVL**	**0.642**	**0.273**	**0.104**	**1.178**
	Pond:Male:SVL	0.111	0.222	−0.325	0.547
Claw	Intercept	−24.483	0.400	−25.301	−23.697
	**Ditch**	**−0.759**	**0.312**	**−1.376**	**−0.145**
	Lake	0.380	0.327	−0.270	1.017
	**Pond**	**0.983**	**0.342**	**0.319**	**1.664**
	**SVL**	**−0.463**	**0.149**	**−0.757**	**−0.170**
	Ditch:Male	−0.147	0.185	−0.511	0.218
	Lake:Male	0.301	0.226	−0.142	0.744
	Pond:Male	−0.098	0.221	−0.531	0.335
	Ditch:SVL	−0.374	0.219	−0.805	0.057
	Lake:SVL	0.480	0.275	−0.062	1.020
	Pond:SVL	0.243	0.254	−0.257	0.742
	Male:SVL	−0.200	0.146	−0.488	0.087
	Ditch:Male:SVL	−0.402	0.214	−0.822	0.017
	**Lake:Male:SVL**	**0.680**	**0.277**	**0.135**	**1.224**
	Pond:Male:SVL	0.294	0.256	−0.209	0.797

Bold values indicate parameters significantly different from zero.

**TABLE 2:** Posterior estimates (mean, SD, and 95% credibility interval) from spatial hierarchical Bayesian best models relating sex, snout-vent length (SVL), and habitat effects to δ^15^N values of *Caiman crocodilus* across landscapes in the Araguaia floodplain.

Tissue	Parameter	Mean	SD	Q_0.025_	Q_0.975_
Plasma	Intercept	6.400	0.218	5.960	6.837
	Ditch	0.273	0.143	−0.011	0.550
	**Pond**	**−0.537**	**0.152**	**−0.834**	**−0.236**
	SVL	0.080	0.068	−0.053	0.213
	Ditch:Male	−0.046	0.081	−0.206	0.114
	Pond:Male	0.105	0.093	−0.078	0.287
	Ditch:SVL	−0.154	0.100	−0.349	0.042
	Lake:SVL	0.096	0.109	−0.118	0.310
	Pond:SVL	0.215	0.116	−0.012	0.442
	Male:SVL	0.061	0.067	−0.071	0.192
	Ditch:Male:SVL	−0.182	0.098	−0.374	0.009
	Lake:Male:SVL	0.132	0.117	−0.096	0.362
	Pond:Male:SVL	0.219	0.116	−0.009	0.446
Muscle	Intercept	7.024	0.197	6.632	7.417
	Ditch	0.228	0.118	−0.005	0.458
	**Lake**	**−0.252**	**0.121**	**−0.489**	**−0.012**
	Pond	−0.214	0.122	−0.454	0.025
	Male	−0.053	0.052	−0.156	0.050
	SVL	0.040	0.053	−0.064	0.143
	Ditch:Male	−0.135	0.085	−0.302	0.031
	Lake:Male	0.152	0.101	−0.046	0.349
	Pond:Male	0.047	0.100	−0.150	0.244
	**Ditch:SVL**	**−0.293**	**0.094**	**−0.478**	**−0.108**
	Lake:SVL	0.181	0.127	−0.068	0.431
	**Pond:SVL**	**0.329**	**0.114**	**0.104**	**0.554**
	**Ditch:Male:SVL**	**−0.194**	**0.090**	**−0.370**	**−0.017**
	Lake:Male:SVL	0.132	0.126	−0.116	0.380
	**Pond:Male:SVL**	**0.262**	**0.116**	**0.034**	**0.490**
Claw	Intercept	7.062	0.182	6.695	7.421
	Ditch	0.119	0.147	−0.172	0.408
	Pond	−0.305	0.160	−0.620	0.008
	Male	−0.032	0.061	−0.151	0.087
	SVL	0.095	0.061	−0.025	0.214
	Ditch:Male	−0.104	0.096	−0.293	0.083
	Lake:Male	0.160	0.113	−0.062	0.382
	Pond:Male	0.026	0.112	−0.196	0.246
	**Ditch:SVL**	**−0.279**	**0.107**	**−0.490**	**−0.069**
	Lake:SVL	0.246	0.144	−0.036	0.528
	Pond:SVL	0.237	0.129	−0.016	0.489
	**Ditch:Male:SVL**	**−0.315**	**0.101**	**−0.514**	**−0.117**
	Lake:Male:SVL	0.224	0.138	−0.047	0.496
	Pond:Male:SVL	0.258	0.131	0.000	0.514

Bold values indicate parameters significantly different from zero.

**TABLE 3:** Posterior estimates (mean, SD, and 95% credibility interval) from spatial hierarchical Bayesian best models relating Boruta-selected landscape attributes to isotopic niche width (SEA_B_) of *Caiman crocodilus* across landscapes in the Araguaia floodplain.

Tissue	Parameter	Mean	SD	Q_0.025_	Q_0.975_
Plasma	Intercept	17.966	7.972	−3.315	33.467
	**PCLASS (Pasture) 0.5-B**	**11.54**	**5.199**	**1.236**	**21.759**
	PCLASS (Pasture) 3-B	−3.205	5.234	−13.511	7.144
Muscle	Intercept	16.833	10.301	−12.355	36.336
	PCLASS (Pasture) 3-B	4.413	2.682	−0.94	9.669
	LDI 3-B	0.724	2.68	−4.523	6.074
Claw	Intercept	19.395	7.557	−1.691	33.682
	PCLASS (Pasture) 3-B	4.478	2.424	−0.323	9.254

Bold values indicate parameters significantly different from zero. Buffers zones for landscape metrics include 0.5-B for 500 m buffer, 1-B for 1 km buffer, and 3-B for 3 km buffer.

## Data Availability

The raw data supporting the conclusions of this article will be made available by the authors, without undue reservation.

## Footnotes

1
http://mapbiomas.org

2
https://semarh.to.gov.br/car/base-vetorial-digital-tematica-do-car/

3
http://www.inpe.br/
