# Supplementary material for: Agricultural input modifies trophic niche and basal energy source of a top predator across human-modified landscapes
Source: Front Ecol Evol. Author manuscript; Available in PMC 2025 Aug 6. (PMC12327407; doi:10.3389/fevo.2023.1053535)
Supplement: Supplement [file NIHMS2033107-supplement-Supplement.pdf]

Supplementary Material

1. Appendix A – Preparation and measurement procedures for amino acid isotope analysis

Following established protocols (Whiteman et al., 2019), an aliquot of ~1-2 mg from each lipid-extracted muscle sample was hydrolyzed to constituent amino acids in 1mL of 6 N hydrochloric acid (HCl) at 110 °C for 20 hours; tubes were flushed with N_2 gas for 30 s and sealed before hydrolysis to prevent oxidation. After, all samples were transferred into 4 mL vials and dried down under a steam of N_2 gas for 1 hour at 110 °C in a Thermo Scientific® Reacti-Therm heating module. This process converted the sample in a solid film in the vials that consisted of pure amino acids (Whiteman et al., 2019). To transform samples into more volatile forms, amino acids were derivatized to N-trifluoroacetic acid isopropyl esters using previously described methods (Silfer et al., 1991; Whiteman et al., 2019). In brief, samples were initially reacted with 1 mL of a 4:1, 2-propanol:acetyl chloride solution for an hour at 110 °C. Samples were then dried down under N_2 gas at room temperature and rinsed twice with dichloromethane (DCM). Lastly, samples were acetylated by adding a 1:1 trifluoroacetic anhydride:dichloromethane solution and reacting at 110 °C for 10 min.

The $\delta^{13}C$ values of individual derivatized amino acids were measured in duplicate using a GC-C-IRMS system. Derivatized samples were injected into a 60-m BPX5 gas chromatography column for amino acid separation (0.32 ID, 1.0 µm film thickness, SGE Analytical Science, Ringwood, Victoria, Australia) in a Thermo Scientific Trace 1310 gas chromatography, coupled to an IsoLink combustion interface attached to a Thermo Scientific Delta Plus IRMS (Bremen, Germany). For amino acid $\delta^{13}C$ measurements, commercially available powdered amino acids (Sigma Aldrich, St. Louis, MO) were used as a primary reference material (PRM) that were derivatized and analyzed alongside each batch of unknown samples. $\delta^{13}C$ values for each underivatized (powdered) amino acid have been previously measured via EA-IRMS in the UNM-CSI. The within-run standard deviations of measured $\delta^{13}C$ values among essential amino acids in the in-house reference material ranged from 0.2‰ (isoleucine) to 0.8‰ (lysine).

Measured $\delta^{13}C$ values of reference materials and unknown samples include carbon from reagents (isopropanol and N-trifluoroacetic acid anhydride) added during derivatization. By derivatizing and analyzing reference materials alongside unknown samples, we can calculate the $\delta^{13}C$ value of the intrinsic amino acid (δXAA_{sample}) using the following equation: $\delta XAA_{sample} = \delta XAA_{dsa} - \delta XAA_{dst} + \delta XAA_{std}\ (p_{std})\ /\ p_{std}$, where δX is the isotope of interest ($\delta^{13}C$), δXAA_{dsa} and δXAA_{dst} refer to the derivatized sample and standard respectively, δXAA_{std} refers to the underivatized standard, and p_{std} is equal to the proportion of the carbon derivative that was sourced from the amino acid (Silfer et al., 1991; O'Brien et al., 2002).

2. Appendix B – Spatial hierarchical Bayesian models with INLA and SPDE

Geostatistical data are realizations of a stochastic process indexed by space (*random field*)

$$Y(s) \equiv \{y(\boldsymbol{s}), \boldsymbol{s} \in \mathcal{D}\},$$

where *random field* is characterized by a spatial index **s** which varies continuously in the fixed domain $\mathcal{D}$ (Blangiardo and Cameletti, 2015; Krainski et al., 2018; Gómez-Rubio, 2020). Typically, **s** is a two-dimensional vector, such as latitude and longitude. In this sense, the actual data are represented by a collection of observations $\mathbf{y} = \{y(\mathbf{s}_1),\ldots, y(\mathbf{s}_n)\}$, where the set of n points $(\mathbf{s}_1,\ldots, \mathbf{s}_n)$ indicate the locations at which the measurements are taken. Such spatial process, a latent Gaussian Random Field (GRF), follows a multivariate Normal distribution with mean $\boldsymbol{\mu} = \{\mu(\mathbf{s}_1),\ldots, \mu(\mathbf{s}_n)\}$ and spatially structured covariance matrix $\Sigma = \{C(\mathbf{s}_1, \mathbf{s}_n)\}$, which is related to the Matérn spatial covariance function (Rue et al., 2009; Lindgren et al., 2011; Blangiardo and Cameletti, 2015; Rue et al., 2017). Additionally, the latent GRF must assume a Gaussian form with additional conditional independence properties, which reflects to a Gaussian Markov Random Fields (GMRF) form, with huge computational benefits because pairs of conditionally independent values result in zeros in the matrix; thus, this implies great importance in Bayesian inferential methods based on Integrated Nested Laplace Approximations – INLA (Rue et al., 2017). The Stochastic Partial Differential Equations (SPDE) approach is implemented by constructing a continuously indexed approximation of the latent GRF defined on the entire study area through a discretely indexed spatial random process (i.e., a GMRF) associated to use of the Matérn covariance function, where the parameters used in SPDE have one-to-one correspondence (Lindgren et al., 2011; Blangiardo and Cameletti, 2015; Bakka et al., 2018).

In INLA, the spatial hierarchical Bayesian model has a structure similar to the Generalized Linear Mixed Model framework, where the response (η) is a linear predictor and the measured parameter, where observations have an associated likelihood. The models estimate the mean (μ) of the observed data using a structured additive predictor η through a link function g(.), such that $g(\mu_i) = \eta$, for example:

$$y_i \sim Normal(\mu_i, \sigma_e^2) \quad i = 1, \ldots, n$$

$$g(\mu_i) = \eta$$

$$\eta_i = \beta_0 + \sum_j \beta_j \, z_{ij} + \xi_i + \varepsilon_i$$

where: σ_e^2 is the hyperparameter of variance of the measurement error e_i which is supposed to be normally distributed and independent; β_0 is the intercept; z are fixed covariates with linear effects; β_j are the linear regression coefficients of the z predictors; the term ξ_i represents the random effect, which is the realization of the latent GF using a Gaussian multivariate distribution $\xi(\mathbf{s}) \sim$ MVNormal(0, Σ), where the mean is zero and the precision matrix Σ relates to Matérn spatial covariance function and by which captures the spatial correlation through mesh structures; finally, ε_i is an error term (Blangiardo et al., 2013; Rue et al., 2017; Krainski et al., 2018; Gómez-Rubio, 2020). All these components constitute the (non-observable) latent field defined as $\theta = \{\beta_0, \beta_j, \xi\}$, where β and ξ are the covariates and smooth functions included in the linear predictor. In the SPDE approach, the mesh structures are representations of latent fields of domain $\mathcal{D}$ in triangular subdivisions of mosaic with indexed node (vertices), and thus, used to construct observation matrix A that connects the GMRF-on-the-mesh to the GMRF-on-the-data (Lindgren and Rue, 2015; Bakka et al., 2018).

Then, we performed the spatial hierarchical Bayesian models where the responses were bulk isotopic compositions (δ^{13}C and δ^{15}N values) from each individual and isotopic niche width (SEA_B) at each site across landscapes in the Araguaia floodplain. Each sampled tissue had separated model, where the predictors for δ^{13}C and δ^{15}N models were sex, snout-vent length (SVL), habitat, and their interactions; otherwise, the SEA_B model had the previously selected by Boruta, which were B_500m_PCLASS_Pasture, B_3km_PCLASS_Pasture, and B_3km_LDI (Table S2). Following Blangiardo and Cameletti (2015), we constructed three models to test for each response: (i) null model; (ii) full model without accounting for spatial process; (iii) full model accounting for spatial process. Overall, the simplified models tested were:

1. δ^{13}C (all tissues):
    i. Null

$$= 1$$

    ii. Non-SPDE

$$= \beta_0 + \beta_1 Habitat + \beta_2 Sex + \beta_3 SVL + \beta_4 Habitat{:}Sex + \beta_5 Habitat{:}SVL + \beta_6 Habitat{:}Sex{:}SVL$$

    iii. SPDE

$$= \beta_0 + \beta_1 Habitat + \beta_2 Sex + \beta_3 SVL + \beta_4 Habitat{:}Sex + \beta_5 Habitat{:}SVL + \beta_6 Habitat{:}Sex{:}SVL + \xi$$

2. δ^{15}N (all tissues):
    i. Null

$$= 1$$

    ii. Non-SPDE

$$= \beta_0 + \beta_1 Habitat + \beta_2 Sex + \beta_3 SVL + \beta_4 Habitat{:}Sex + \beta_5 Habitat{:}SVL + \beta_6 Habitat{:}Sex{:}SVL$$

    iii. SPDE

$$= \beta_0 + \beta_1 Habitat + \beta_2 Sex + \beta_3 SVL + \beta_4 Habitat{:}Sex + \beta_5 Habitat{:}SVL + \beta_6 Habitat{:}Sex{:}SVL + \xi$$

3. SEA_B:
    a. Plasma and Muscle
        i. Null

$$= 1$$

        ii. Non-SPDE

$$= \beta_0 + \beta_1\ B\_500m\_PCLASS\_Pasture + \beta_2\ B\_3km\_PCLASS\_Pasture + \beta_3\ B\_3km\_LDI$$

        iii. SPDE

$$= \beta_0 + \beta_1 \, B\_500m\_PCLASS\_Pasture + \beta_2 \, B\_3km\_PCLASS\_Pasture + \beta_3 \, B\_3km\_LDI + \xi$$

b. Claw

i. Null

$$= 1$$

ii. Non-SPDE

$$= \beta_0 + \beta_1 \, B\_3km\_PCLASS\_Pasture$$

iii. SPDE

$$= \beta_0 + \beta_1 \, B\_3km\_PCLASS\_Pasture + \xi$$

However, we obtained the best model designed from these previous models using a backward stepwise procedure in the *INLAstep* function from R package INLAUTILS (Redding et al., 2017). The best models are reported in the Table 1-3 of main text. For these best models, hyperparameters estimated are given in Supplementary Table 8.

2.1. References

Amelung, W., and Zhang, X. (2001). Determination of amino acid enantiomers in soils. *Soil Biology and Biochemistry* 33(4), 553-562. doi: https://doi.org/10.1016/S0038-0717(00)00195-4.

Bakka, H., Rue, H., Fuglstad, G.-A., Riebler, A., Bolin, D., Illian, J., et al. (2018). Spatial modeling with R-INLA: A review. *Wiley Interdisciplinary Reviews: Computational Statistics* 10(6). doi: 10.1002/wics.1443.

Blangiardo, M., and Cameletti, M. (2015). *Spatial and spatio-temporal Bayesian models with R-INLA.* Chichester: John Wiley & Sons Ltd.

Blangiardo, M., Cameletti, M., Baio, G., and Rue, H. (2013). Spatial and spatio-temporal models with R-INLA. *Spat Spatiotemporal Epidemiol* 7, 39-55. doi: 10.1016/j.sste.2013.07.003.

Gómez-Rubio, V. (2020). *Bayesian inference with INLA.* London: Chapman and Hall/CRC Press.

Krainski, E.T., Gómez-Rubio, V., Bakka, H., Lenzi, A., Castro-Camilo, D., Simpson, D., et al. (2018). *Advanced spatial modeling with stochastic partial differential equations using R and INLA.* London: Chapman and Hall/CRC Press.

Lindgren, F., and Rue, H. (2015). Bayesian spatial modelling with R-INLA. *Journal of Statistical Software* 63(19), 25. doi: 10.18637/jss.v063.i19.

Lindgren, F., Rue, H., and Lindström, J. (2011). An explicit link between Gaussian fields and Gaussian Markov random fields: The stochastic partial differential equation approach. *Journal of the Royal Statistical Society: Series B (Statistical Methodology)* 73(4), 423-498. doi: https://doi.org/10.1111/j.1467-9868.2011.00777.x.

O'Brien, D.M., Fogel, M.L., and Boggs, C.L. (2002). Renewable and nonrenewable resources: Amino acid turnover and allocation to reproduction in Lepidoptera. *Proc Natl Acad Sci U S A* 99(7)**,** 4413-4418. doi: 10.1073/pnas.072346699.

Redding, D.W., Lucas, T.C.D., Blackburn, T.M., and Jones, K.E. (2017). Evaluating Bayesian spatial methods for modelling species distributions with clumped and restricted occurrence data. *PLOS ONE* 12(11)**,** e0187602. doi: 10.1371/journal.pone.0187602.

Rue, H., Martino, S., and Chopin, N. (2009). Approximate Bayesian inference for latent Gaussian models by using integrated nested Laplace approximations. *Journal of the Royal Statistical Society: Series B (Statistical Methodology)* 71(2)**,** 319-392. doi: 10.1111/j.1467-9868.2008.00700.x.

Rue, H., Riebler, A., Sørbye, S.H., Illian, J.B., Simpson, D.P., and Lindgren, F.K. (2017). Bayesian computing with INLA: A review. *Annual Review of Statistics and Its Application* 4**,** 395-421.

Silfer, J.A., Engel, M.H., Macko, S.A., and Jumeau, E.J. (1991). Stable carbon isotope analysis of amino acid enantiomers by conventional isotope ratio mass spectrometry and combined gas chromatography/isotope ratio mass spectrometry. *Analytical Chemistry* 63(4)**,** 370-374. doi: 10.1021/ac00004a014.

Whiteman, J.P., Smith, E.A.E., Besser, A.C., and Newsome, S.D. (2019). A guide to using compound-specific stable isotope analysis to study the fates of molecules in organisms and ecosystems. *Diversity-Basel* 11(1)**,** 18. doi: 10.3390/d11010008.

3. Appendix C – Linear discriminant analysis output for habitat categorization of essential amino acids analysis ($\delta^{13}C_{EAA}$)

Prior probabilities of groups

Ditch	Lake	Pond	River
0.25	0.25	0.25	0.25

Group means

Habitat	Thr	Ile	Leu	Val	Lys	Phe	Tyr
Ditch	-17.0	-24.8	-31.0	-27.5	-23.8	-28.9	-28.9
Lake	-16.1	-24.0	-30.0	-27.4	-23.2	-30.9	-30.4
Pond	-17.3	-24.0	-29.9	-26.4	-22.3	-27.9	-28.2
River	-19.0	-25.6	-31.7	-28.5	-23.9	-32.4	-31.5

Coefficients of linear discriminants

Parameter	LD1	LD2	LD3
Thr	0.108	-0.622	0.306
Ile	0.149	0.375	-0.220
Leu	0.793	-1.325	-0.686
Val	-0.105	1.063	0.017
Lys	0.798	0.708	-0.125
Phe	-1.721	-0.147	-0.259
Tyr	-0.214	-0.066	0.466

Proportion of trace

LD1	LD2	LD3
0.8856	0.0922	0.0222

Linear discriminant analysis (LDA) classification table.

	Ditch	Lake	Pond	River	Percent correct
Ditch	**6**	0	4	0	60
Lake	0	**7**	0	3	70
Pond	4	0	**5**	1	50
River	0	2	0	**8**	80

Bold numbers represent correct reclassification of the LDA.

4. Appendix D – Linear discriminant analysis output for locality categorization of essential amino acids analysis ($\delta^{13}C_{EAA}$)

Prior probabilities of groups

Bananal	Canguçu	Cooperformoso	Coopergran
0.25	0.25	0.25	0.25

Group means

Locality	Thr	Ile	Leu	Val	Lys	Phe	Tyr
Bananal	-17.0	-25.1	-30.9	-27.3	-23.4	-31.6	-30.8
Canguçu	-18.0	-24.5	-30.8	-28.6	-23.8	-31.7	-31.1
Cooperformoso	-16.3	-23.8	-30.1	-26.1	-23.3	-28.2	-29.0
Coopergran	-18.0	-25.0	-30.8	-27.7	-22.9	-28.6	-28.0

Coefficients of linear discriminants

Parameter	LD1	LD2	LD3
Thr	-0.164	0.406	0.320
Ile	-0.137	-1.103	-0.735
Leu	-0.957	-0.987	-0.171
Val	0.265	1.160	-0.692
Lys	-0.700	0.698	0.689
Phe	1.688	-0.152	-0.238
Tyr	0.192	-0.159	0.560

Proportion of trace

LD1	LD2	LD3
0.8024	0.1094	0.0881

Linear discriminant analysis (LDA) classification table

	Bananal	Canguçu	Cooperformoso	Coopergran	Percent correct
Bananal	**10**	0	0	0	100
Canguçu	3	**7**	0	0	70
Cooperformoso	0	0	**9**	1	90
Coopergran	0	0	2	**8**	80

Bold numbers represent correct reclassification of the LDA.

5. Supplementary Figures and Tables

5.1. Supplementary Figures

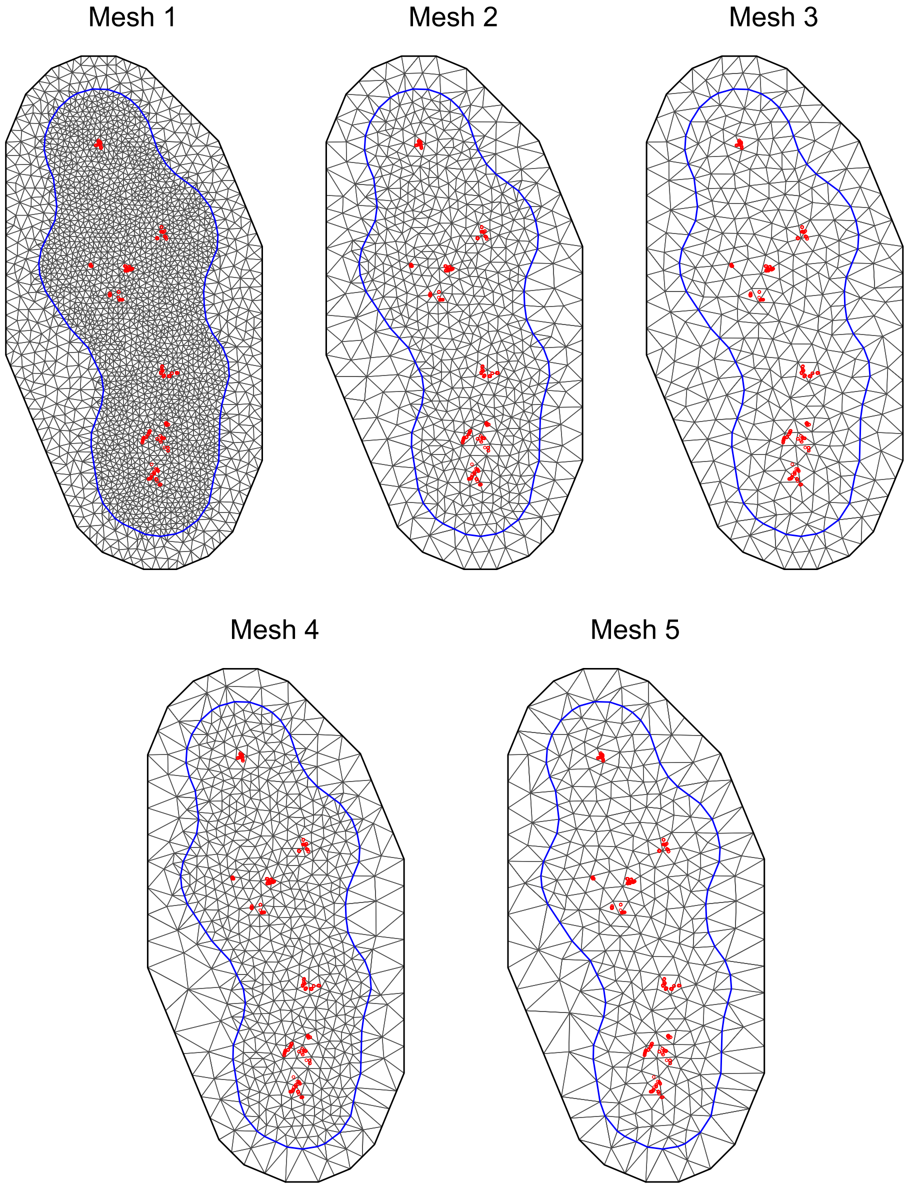

Supplementary Figure 1. Meshes used in the SPDE approach to INLA model selection. First, we constructed five meshes with a specified non-convex boundary (convex = -0.15) with *inla.nonconvex.hull* function. Next, we built non-convex meshes using the *inla.mesh.2d* function with fixed values for *cutoff* and *offset* parameters (*cutoff* = 0.01 and *offset* = (0.1, 0.2)) and varying values of the *max edge* parameter: Mesh I (*max edge* = (0.05, 0.1)), Mesh II (*max edge* = (0.1, 0.2)), Mesh III (*max edge* = (0.15, 0.2)) and Mesh IV (*max edge* = (0.1, 0.3)), and Mesh V (*max edge* = (0.15, 0.3)).

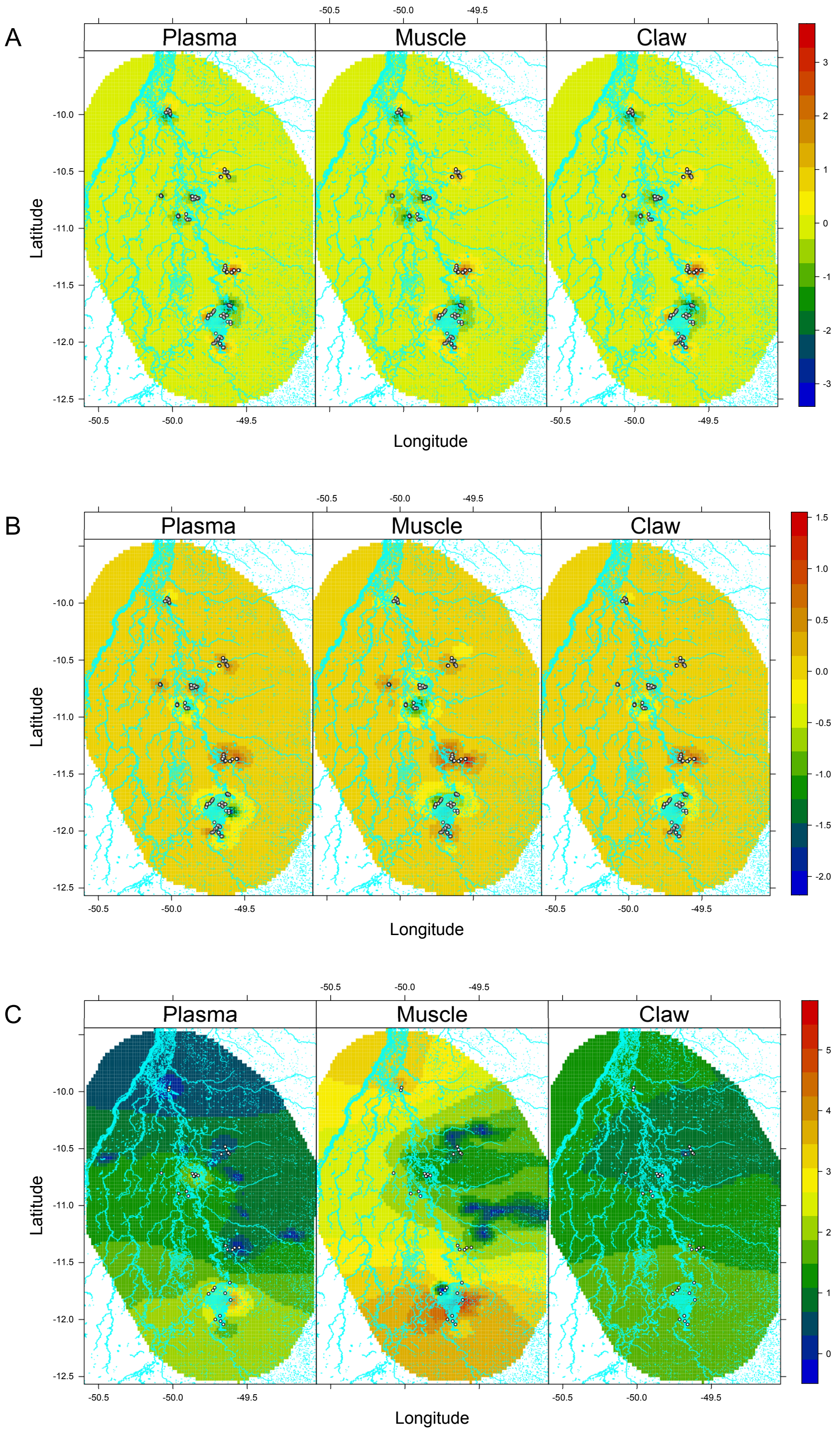

Supplementary Figure 2. Posterior mean of the spatial random effect, Gaussian random field, from spatial hierarchical Bayesian best models for (A) $\delta^{13}C$, (B) $\delta^{15}N$, and (C) isotopic niche width according to the tissue of *Caiman crocodilus* across landscapes in the Araguaia floodplain. Points represent individuals (for A and B) and sites (C) within a determined locality. The colors indicate levels of spatial random effect according to the associated legends.

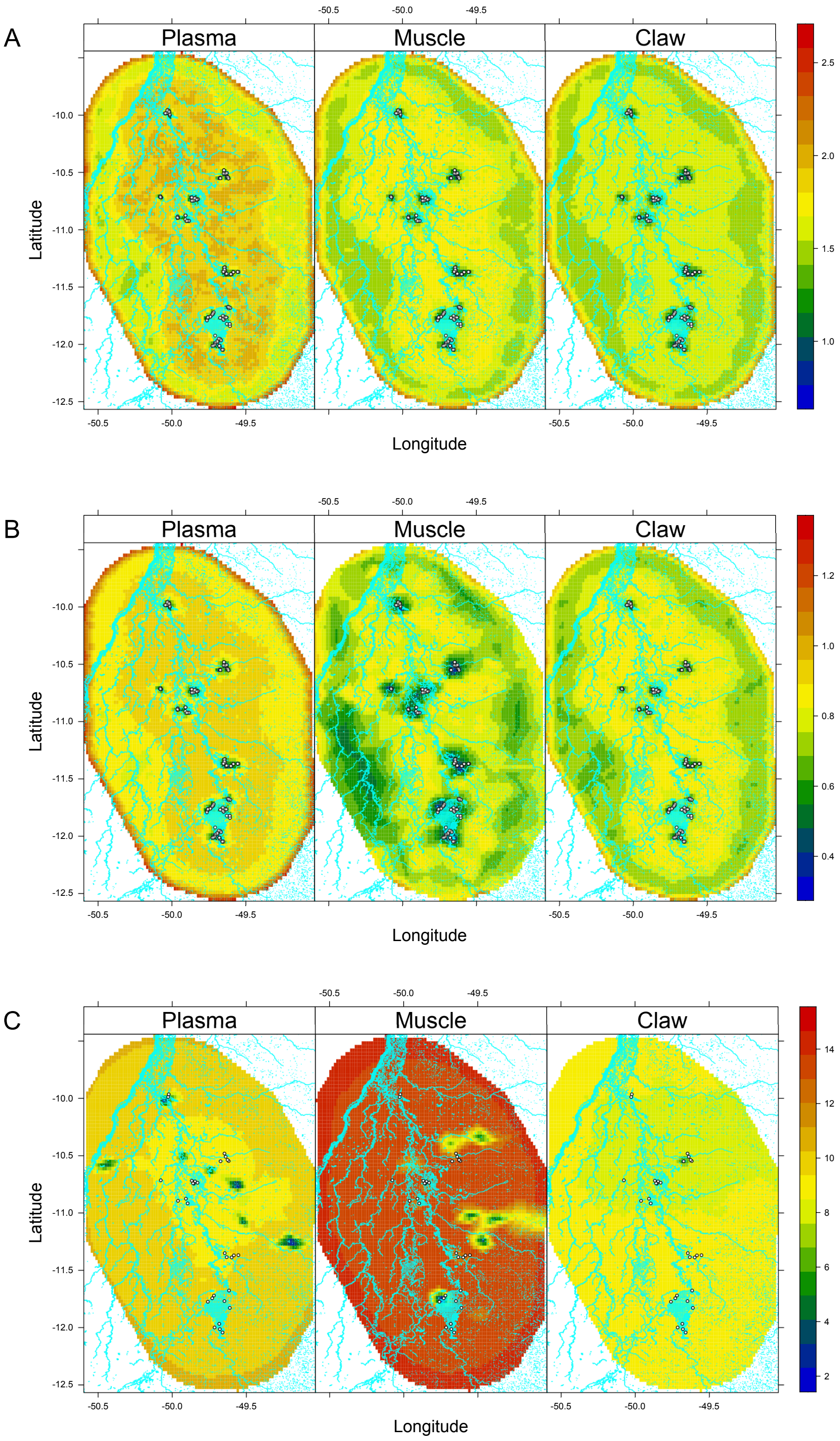

Supplementary Figure 3. Posterior standard deviation of the spatial random effect, Gaussian random field, from spatial hierarchical Bayesian best models for (A) δ^{13}C, (B) δ^{15}N, and (C) isotopic niche width according to the tissue of *Caiman crocodilus* across landscapes in the Araguaia floodplain. Points represent individuals (for A and B) and sites (C) within a determined locality. The colors indicate levels of spatial random effect according to the associated legends.

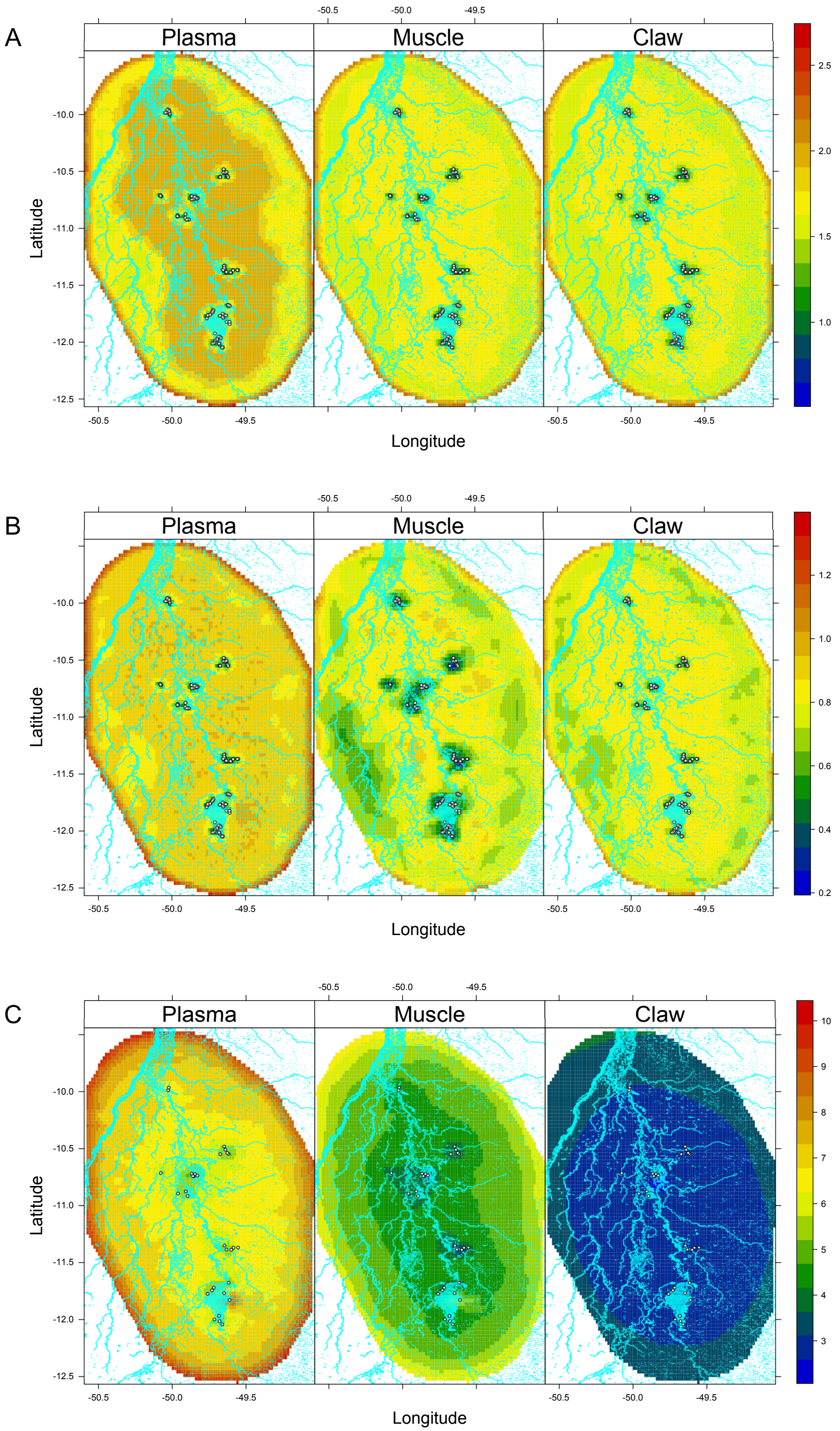

Supplementary Figure 4. Predicted standard deviation of the spatial hierarchical Bayesian best models for (A) δ^{13}C, (B) δ^{15}N, and (C) isotopic niche width according to the tissue of *Caiman crocodilus* across landscapes in the Araguaia floodplain. Points represent individuals (for A and B) and sites (C) within a determined locality. The colors indicate levels of responses according to the associated legends.

5.2. Supplementary Tables

Supplementary Table 1. Landscape attributes retained by the Variance Inflation Factor (VIF) in the Araguaia floodplain. The VIF threshold had a value of 4 for *theta* parameter. NS: landscape attributes with collinearity problem (VIF value > 4) and that were excluded from the posterior analysis. Buffers zones for landscape metric indicated through 0.5-B for 500-m buffer, 1-B for 1-km buffer, and 3-B for 3-km buffer.

Variables	VIF
PCLASS (Forest) 0.5-B	3.61
PCLASS (Savanna) 0.5-B	2.19
PCLASS (Pasture) 0.5-B	2.51
PCLASS (Water) 0.5-B	2.99
PCLASS (Forest) 3-B	2.73
PCLASS (Pasture) 3-B	2.94
MPA (Water) 1-B	2.37
MPA (Water) 3-B	1.56
ENN (Water) 1-B	1.25
ENN (Water) 3-B	1.59
COE (Water) 0.5-B	1.92
COE (Water) 3-B	2.64
LDI 0.5-B	3.24
LDI 3-B	1.78
PCLASS (Crop) 1-B	NS
LPI (Water) 1-B	NS
PCLASS (Crop) 3-B	NS
LPI (Water) 0.5-B	NS
PCLASS (Water) 3-B	NS
PCLASS (Crop) 0.5-B	NS
PCLASS (Water) 1-B	NS
PCLASS (Savanna) 1-B	NS
PCLASS (Pasture) 1-B	NS
PCLASS (Forest) 1-B	NS
LDI 1-B	NS
COE (Water) 1-B	NS
MPA (Water) 0.5-B	NS
PCLASS (Savanna) 3-B	NS
LPI (Water) 3-B	NS

Supplementary Table 2. Detailed population structure of *Caiman crocodilus* and landscape metric selected by Boruta in the Araguaia floodplain.

Locality	Number of sites	Habitat	N	Sex ratio (Male:Female)	SVL (cm)		Buffer 500 m PCLASS Pasture (%)		Buffer 3 km PCLASS Pasture (%)		Buffer 3 km LDI	
					Mean	SD	Mean	SD	Mean	SD	Mean	SD
Bananal	2	Ditch	12	7:5	58.17	17.09	0.13	0.35	0.30	0.04	0.48	0.09
	1	Lake	12	7:5	65.72	17.75	0.00	0.00	0.00	0.00	0.33	0.02
	1	River	12	9:3	62.90	21.52	0.11	0.39	0.31	0.05	0.86	0.02
Canguçu	1	Lake	9	4:5	62.90	15.53	11.59	15.15	25.85	8.80	0.57	0.12
	1	River	12	12:0	64.88	16.85	9.13	13.86	15.81	11.98	0.74	0.12
Cooperformoso	1	Ditch	10	7:3	69.79	25.22	0.35	0.63	4.53	3.48	0.67	0.06
	2	Lake	11	9:2	59.40	13.21	20.76	29.63	39.76	16.10	0.67	0.09
	1	Pond	12	6:6	56.77	15.78	60.34	9.07	38.62	0.37	0.81	0.01
	1	River	12	10:2	57.34	16.96	28.49	16.89	54.33	22.80	0.78	0.05
Coopergran	1	Ditch	12	9:3	57.12	19.05	0.00	0.00	4.25	4.70	0.27	0.26
	2	Lake	14	11:3	71.58	21.13	15.05	16.80	29.65	5.07	0.84	0.04
	2	Pond	10	7:3	55.61	14.67	38.15	8.31	26.61	6.58	0.72	0.04
	1	River	13	12:1	67.52	22.99	57.11	13.92	51.32	4.90	0.67	0.01
Cristalândia	2	Ditch	12	6:6	59.79	8.69	0.00	0.00	17.80	26.18	0.70	0.01
	1	Lake	10	8:2	59.29	13.53	31.46	5.40	50.56	1.52	0.86	0.00
	1	Pond	6	3:3	59.63	20.81	29.44	9.82	48.75	3.38	0.80	0.06
	1	River	11	11:0	57.37	10.33	7.54	7.54	27.54	3.53	0.76	0.02
Lagoa	1	Ditch	13	6:7	47.56	21.41	1.04	2.42	18.94	2.67	0.89	0.01
	1	Lake	7	2:5	56.91	17.27	26.28	9.10	19.98	1.08	0.88	0.02
	1	Pond	8	4:4	48.11	16.88	59.64	24.80	23.04	0.07	0.86	0.01
	2	River	14	8:7	68.54	14.99	13.99	15.35	18.84	2.77	0.84	0.04
Xavante	1	Ditch	12	1:1	62.57	14.52	0.84	1.39	3.25	3.12	0.64	0.08
	1	Lake	10	9:1	58.34	10.21	20.63	10.18	43.36	1.32	0.77	0.01
	2	Pond	11	7:4	46.14	12.65	59.84	10.78	37.64	0.26	0.71	0.08
	1	River	10	4:6	60.92	7.79	18.07	2.50	25.82	0.38	0.79	0.01

Supplementary Table 3. Information of the 40 *Caiman crocodilus* selected for carbon essential amino acids ($\delta^{13}C_{EAA}$) analyzes.

Locality	Habitat	N	Sex ratio (Male:Female)	SVL (cm)	
				Mean	SD
Bananal	Lake	5	3:2	60.54	18.96
	River	5	3:2	63.16	20.41
Canguçu	Lake	5	5:0	69.02	18.47
	River	5	3:2	62.78	26.26
Cooperformoso	Ditch	5	3:2	73.90	24.44
	Pond	5	3:2	62.54	16.72
Coopergran	Ditch	5	3:2	67.12	24.39
	Pond	5	2:3	62.74	15.24

Supplementary Table 4. Summary of parameters of Boruta selection for isotopic niche width (SEA_B) according to the tissue of *Caiman crocodilus* related to landscape attributes measured in the Araguaia floodplain: mean, minimum, maximum, and normalized importance based on the fraction of random forest runs in which the attributes were more important than shadow values. Buffers zones for landscape metric indicated through 0.5-B for 500-m buffer, 1-B for 1-km buffer, and 3-B for 3-km buffer.

Tissue	Predictor	*meanImp*	*minImp*	*maxImp*	*normHits*
Plasma	**PCLASS (Pasture) 0.5-B**	**18.545**	**11.571**	**23.735**	**0.974**
	**PCLASS (Pasture) 3-B**	**7.665**	**2.483**	**12.249**	**0.652**
	**LDI 3-B**	**0.703**	**-2.080**	**3.127**	**0.035**
	COE (Water) 3-B	-0.708	-3.312	1.656	0.000
	PCLASS (Water) 0.5-B	-0.752	-2.167	1.151	0.000
	LDI 0.5-B	-0.924	-3.708	2.202	0.009
	COE (Water) 0.5-B	-1.365	-3.226	1.502	0.000
	PCLASS (Forest) 0.5-B	-1.571	-2.938	0.256	0.000
	MPA (Water) 3-B	-1.816	-5.548	1.548	0.009
	ENN (Water) 3-B	-2.140	-3.918	-0.161	0.000
	MPA (Water) 1-B	-2.218	-2.805	-0.928	0.000
	ENN (Water) 1-B	-2.243	-3.796	-0.289	0.000
	PCLASS (Forest) 3-B	-2.431	-4.442	-0.998	0.000
	PCLASS (Savanna) 0.5-B	-2.608	-3.835	-1.052	0.000
Muscle	**PCLASS (Pasture) 3-B**	**7.077**	**0.224**	**14.523**	**0.476**
	**LDI 3-B**	**3.586**	**0.704**	**9.267**	**0.001**
	**PCLASS (Pasture) 3-B**	**2.978**	**-0.923**	**5.982**	**0.001**
	PCLASS (Forest) 0.5-B	0.147	-1.861	2.622	0.000
	PCLASS (Forest) 3-B	-0.084	-2.571	1.718	0.000
	ENN (Water) 3-B	-0.839	-2.176	1.453	0.000
	LDI 0.5-B	-1.086	-2.633	0.209	0.000
	PCLASS (Water) 0.5-B	-1.087	-3.138	0.187	0.000
	ENN (Water) 1-B	-1.569	-3.499	0.400	0.000
	PCLASS (Savanna) 0.5-B	-1.739	-4.086	0.889	0.000
	MPA (Water) 3-B	-2.571	-4.714	-1.136	0.000
	COE (Water) 0.5-B	-3.010	-5.122	-0.803	0.000
	MPA (Water) 1-B	-3.702	-4.586	-1.407	0.000
	COE (Water) 3-B	-3.862	-5.526	-1.882	0.000
Claw	**PCLASS (Pasture) 3-B**	**9.619**	**3.059**	**16.860**	**0.612**
	PCLASS (Forest) 3-B	1.126	-1.128	2.265	0.000
	ENN (Water) 3-B	0.080	-2.150	2.238	0.000
	ENN (Water) 1-B	0.039	-1.449	2.182	0.000
	PCLASS (Savanna) 0.5-B	-0.262	-2.620	4.933	0.000
	COE (Water) 0.5-B	-0.519	-4.286	1.562	0.000
	LDI 3-B	-0.740	-1.951	0.661	0.000

PCLASS (Pasture) 0.5-B	-0.865	-1.781	0.764	0.000
PCLASS (Water) 0.5-B	-1.124	-2.577	-0.133	0.000
LDI 0.5-B	-1.620	-4.024	1.341	0.000
PCLASS (Forest) 0.5-B	-1.828	-3.870	0.046	0.000
MPA (Water) 3-B	-2.017	-3.209	-0.672	0.000
MPA (Water) 1-B	-2.358	-4.476	-0.887	0.000
COE (Water) 3-B	-2.362	-4.111	-1.173	0.000

Supplementary Table 5. Accuracy information criteria of mesh designs from different spatial hierarchical Bayesian models for each tissue relating $\delta^{13}C$ values of *Caiman crocodilus* to sex, ontogeny, and habitat across landscapes in the Araguaia floodplain. Bold values indicate mesh design and model chose for posterior analysis. Model descriptions are described in Appendix A.

Tissue	Mesh	DIC				WAIC			
		Null	Non-SPDE	SPDE	Best model	Null	Non-SPDE	SPDE	Best model
Plasma	**Mesh 1**	1215.587	1184.973	1062.336	**1054.824**	1215.735	1186.426	1071.379	**1059.407**
	Mesh 2	1215.587	1184.973	1076.590	1070.672	1215.735	1186.426	1082.268	1072.129
	Mesh 3	1215.587	1184.973	1078.516	1072.786	1215.735	1186.426	1084.668	1074.338
	Mesh 4	1215.587	1184.973	1076.542	1070.820	1215.735	1186.426	1082.216	1072.125
	Mesh 5	1215.587	1184.973	1078.549	1072.897	1215.735	1186.426	1084.700	1074.331
Muscle	**Mesh 1**	1224.719	1189.252	1079.481	**1075.445**	1224.725	1188.863	1086.761	**1080.745**
	Mesh 2	1224.719	1189.252	1088.368	1086.907	1224.725	1188.863	1092.801	1088.798
	Mesh 3	1224.719	1189.252	1088.705	1087.096	1224.725	1188.863	1093.203	1089.531
	Mesh 4	1224.719	1189.252	1088.281	1086.704	1224.725	1188.863	1092.889	1088.659
	Mesh 5	1224.719	1189.252	1088.952	1087.048	1224.725	1188.863	1093.421	1089.500
Claw	**Mesh 1**	1220.159	1185.034	1078.919	**1076.872**	1220.238	1185.340	1085.464	**1081.347**
	Mesh 2	1220.159	1185.034	1088.800	1084.906	1220.238	1185.340	1093.042	1087.417
	Mesh 3	1220.159	1185.034	1087.318	1085.589	1220.238	1185.340	1091.489	1087.377
	Mesh 4	1220.159	1185.034	1088.929	1084.875	1220.238	1185.340	1093.154	1087.503
	Mesh 5	1220.159	1185.034	1087.314	1085.573	1220.238	1185.340	1091.505	1087.545

Supplementary Table 6. Accuracy information criteria of mesh designs from different spatial hierarchical Bayesian models for each tissue relating δ^{15}N values of *Caiman crocodilus* to sex, ontogeny, and habitat across landscapes in the Araguaia floodplain. Bold values indicate mesh design and model chose for posterior analysis. Model descriptions are described in Appendix A.

Tissue	Mesh	DIC				WAIC			
		Null	Non-SPDE	SPDE	Best model	Null	Non-SPDE	SPDE	Best model
Plasma	**Mesh 1**	795.551	762.731	656.718	**648.695**	796.364	765.030	662.052	**652.016**
	Mesh 2	795.551	762.731	657.561	655.927	796.364	765.030	661.620	657.650
	Mesh 3	795.551	762.731	652.109	650.449	796.364	765.030	655.783	652.030
	Mesh 4	795.551	762.731	657.538	652.334	796.364	765.030	661.559	653.666
	Mesh 5	795.551	762.731	652.227	650.473	796.364	765.030	655.958	652.027
Muscle	Mesh 1	733.805	736.360	646.003	643.957	733.691	737.380	647.630	643.734
	Mesh 2	733.805	736.360	648.844	644.890	733.691	737.380	649.672	644.297
	Mesh 3	733.805	736.360	641.496	639.384	733.691	737.380	642.458	638.706
	Mesh 4	733.805	736.360	648.834	644.879	733.691	737.380	649.662	644.304
	**Mesh 5**	733.805	736.360	641.423	**639.375**	733.691	737.380	642.368	**638.660**
Claw	**Mesh 1**	794.461	784.527	709.619	**705.288**	794.469	785.074	713.007	**706.877**
	Mesh 2	794.461	784.527	719.626	715.522	794.469	785.074	720.990	715.024
	Mesh 3	794.461	784.527	709.160	707.033	794.469	785.074	710.657	706.655
	Mesh 4	794.461	784.527	719.641	715.475	794.469	785.074	721.012	715.034
	Mesh 5	794.461	784.527	709.116	707.027	794.469	785.074	710.605	706.667

Supplementary Table 7. Accuracy information criteria of mesh designs from different spatial hierarchical Bayesian models for each tissue relating isotopic niche width (SEA_B) of *Caiman crocodilus* to Boruta-selected landscapes attributes in the Araguaia floodplain. Bold values indicate mesh design and model chose for posterior analysis. Model descriptions are described in Appendix A. NA means that step for model construction was not performed.

Tissue	Mesh	DIC				WAIC			
		Null	Non-SPDE	SPDE	Best model	Null	Non-SPDE	SPDE	Best model
Plasma	Mesh 1	275.724	273.581	273.152	271.674	277.406	274.294	273.625	271.041
	**Mesh 2**	275.724	273.581	273.105	**271.154**	277.406	274.294	273.509	**270.938**
	Mesh 3	275.724	273.581	273.038	271.721	277.406	274.294	273.490	271.278
	Mesh 4	275.724	273.581	273.071	271.580	277.406	274.294	273.441	270.969
	Mesh 5	275.724	273.581	273.073	271.313	277.406	274.294	273.452	270.939
Muscle	Mesh 1	249.977	251.260	250.992	248.951	251.478	251.166	250.850	247.842
	Mesh 2	249.977	251.260	250.880	248.872	251.478	251.166	250.896	247.987
	Mesh 3	249.977	251.260	250.810	248.838	251.478	251.166	250.804	247.971
	Mesh 4	249.977	251.260	250.953	248.819	251.478	251.166	250.719	247.901
	**Mesh 5**	249.977	251.260	250.661	**248.830**	251.478	251.166	250.746	**247.870**
Claw	**Mesh 1**	254.029	252.517	**252.535**	NA	254.453	252.371	**252.477**	NA
	Mesh 2	254.029	252.517	252.658	NA	254.453	252.371	252.629	NA
	Mesh 3	254.029	252.517	252.560	NA	254.453	252.371	252.509	NA
	Mesh 4	254.029	252.517	252.542	NA	254.453	252.371	252.502	NA
	Mesh 5	254.029	252.517	252.572	NA	254.453	252.371	252.528	NA

Supplementary Table 8. Posterior estimates of hyperparameters (mean ± standard deviation and 95% credibility interval) from spatial hierarchical Bayesian best models for $\delta^{13}C$, $\delta^{15}N$, and isotopic niche width (SEA_B) of *Caiman crocodilus* according to tissue across landscapes in the Araguaia floodplain.

Response	Tissue	Hyperparameter	Mean	SD	$Q_{0.025}$	$Q_{0.975}$
bulk $\delta^{13}C$	Plasma	Precision for the Gaussian observations	0.44	0.04	0.36	0.53
		Range for spatial random field	-5.69	0.49	-6.67	-4.76
		SD for spatial random field	3.84	0.43	3.02	4.71
	Muscle	Precision for the Gaussian observations	0.41	0.04	0.34	0.49
		Range for spatial random field	-5.29	0.51	-6.29	-4.28
		SD for spatial random field	3.60	0.47	2.67	4.51
	Claw	Precision for the Gaussian observations	0.41	0.04	0.33	0.49
		Range for spatial random field	-5.18	0.50	-6.17	-4.20
		SD for spatial random field	3.52	0.47	2.61	4.44
bulk $\delta^{15}N$	Plasma	Precision for the Gaussian observations	1.93	0.18	1.59	2.31
		Range for spatial random field	-4.58	0.40	-5.38	-3.81
		SD for spatial random field	3.51	0.41	2.73	4.33
	Muscle	Precision for the Gaussian observations	1.93	0.18	1.60	2.30
		Range for spatial random field	-4.94	0.91	-6.83	-3.27
		SD for spatial random field	3.62	0.57	2.56	4.82
	Claw	Precision for the Gaussian observations	1.57	0.15	1.29	1.89
		Range for spatial random field	-4.81	0.52	-5.84	-3.80
		SD for spatial random field	3.81	0.44	2.94	4.68
SEA_B	Plasma	Precision for the Gaussian observations	0.00	0.00	0.00	0.01
		Range for spatial random field	-1.64	2.43	-5.81	3.68
		SD for spatial random field	2.99	2.27	-0.69	8.05
	Muscle	Precision for the Gaussian observations	0.01	0.00	0.00	0.01
		Range for spatial random field	-3.00	2.54	-7.84	2.12
		SD for spatial random field	1.51	1.93	-2.05	5.50

	Claw	Precision for the Gaussian observations	0.01	0.00	0.00	0.01
		Range for spatial random field	-1.41	2.16	-5.08	3.31
		SD for spatial random field	2.83	2.34	-1.20	7.92
